# Systemic–local immune remodeling in colorectal cancer: CD14+ cell-derived cytokine responsiveness, mucosal immune-cell organization, and exploratory metastasis-associated patterns

**DOI:** 10.3389/fimmu.2026.1918710

**Published:** 2026-09-09

**Authors:** Nikolay K. Shakhpazyan, Liudmila M. Mikhaleva, Nikolay K. Sadykhov, Alexandra K. Konyukova, Arkady L. Bedzhanyan, Zarina V. Gioeva, Marina V. Khrustaleva, Elina A. Godzhello

**Affiliations:** Petrovsky National Research Centre of Surgery, Moscow, Russia

**Keywords:** CD14+ cells, colorectal cancer, IL-1β, IL-6, immune remodeling, LPS stimulation, metastasis

## Abstract

**Background:**

Immune regulatory networks contribute to colorectal cancer (CRC) progression, but most clinical studies focus on tissue immune-cell abundance or static inflammatory mediators. We investigated cytokine-response patterns in cultures derived from peripheral-blood CD14+ cells and local immune-cell composition in tumor-adjacent and resection-margin mucosa.

**Methods:**

This single-center prospective observational study included 106 patients with colorectal adenocarcinoma and 20 comparison subjects with non-malignant surgical conditions. Peripheral-blood CD14+ cells were isolated, differentiated with M-CSF, and subjected to sequential LPS stimulation. IL-1β, IL-6, IL-8, and MCP-1 concentrations were measured, and cytokine-response indices were calculated. In CRC patients, CD4+, CD8+, CD20+, CD56+, and CD68+ cell densities were quantified immunohistochemically in paired mucosal compartments. Analyses addressed CRC-associated differences, local immune-cell organization, systemic–local associations, and an exploratory comparison between patients without distant metastases (n = 97) and patients with distant metastases (n = 9).

**Results:**

CRC was associated with selective remodeling of IL-1β and IL-6 response indices. Compared with the comparison group, CRC patients showed higher IL-1β post-washout persistence, lower shutdown efficiency and re-induction capacity, and a higher re-stimulation index. IL-6 responses were characterized by lower basal activity but higher basal drift, primary LPS response, and post-washout persistence. The exploratory comparison according to distant metastasis status identified additional differences in selected IL-1β and IL-6 indices, however, these findings are limited by the small number of patients with distant metastases. Locally, CD20+ cells were enriched in tumor-adjacent mucosa, whereas CD68+ cells were enriched in resection-margin mucosa. Matched immune-cell densities were positively correlated across compartments. The strongest systemic-local association was an inverse correlation between MCP-1 primary LPS response and CD68+ cell density in tumor-adjacent mucosa.

**Conclusions:**

CRC is associated with altered cytokine responsiveness in M-CSF-differentiated cultures derived from blood CD14+ cells and with compartment-specific mucosal immune-cell organization. The observed systemic–local associations suggest partial coupling between these experimental and tissue-level immune features. Exploratory metastasis-associated patterns were identified but require validation in larger, adequately powered cohorts.

## 1 Introduction

Colorectal cancer (CRC) remains one of the most common malignancies worldwide and is a major cause of cancer-related mortality (1). Beyond the accumulation of tumor-cell genetic and epigenetic alterations, CRC development and progression are strongly shaped by the immune and inflammatory context in which the tumor evolves. The type, density, and spatial distribution of immune cells within colorectal tumors have substantial biological and prognostic relevance, as illustrated by the development and validation of immune scoring approaches based mainly on T-cell infiltration in the tumor center and invasive margin (2–4). These observations indicate that CRC should be considered not only as an epithelial malignancy, but also as a disease involving complex tumor-immune interactions.

Much of the current evidence in CRC immunology has focused on the local tumor microenvironment, including tumor-infiltrating lymphocytes, macrophages, neutrophils, dendritic cells, natural killer cells, myeloid-derived suppressor cells, and their interactions with malignant epithelial cells and stromal components (5–7). Innate immune cells are particularly relevant in this context because they can promote or restrain tumor progression through cytokine and chemokine secretion, antigen presentation, matrix remodeling, angiogenesis, immune suppression, and regulation of adaptive immune responses (8). Among these cells, monocyte and macrophage-lineage populations are highly plastic and may acquire functionally distinct states depending on local and systemic cues. In CRC, tumor-associated macrophages have been implicated in invasion, immune evasion, angiogenesis, metastatic dissemination, and therapeutic resistance (8).

Within the metastatic cascade, such immune regulatory networks are relevant not only as local features of the tumor microenvironment but also as systemic host responses. Myeloid cells, macrophage-lineage populations, and cytokine or chemokine programs can shape tumor invasion, immune evasion, angiogenesis, dissemination, and the establishment of permissive metastatic niches. For gastrointestinal malignancies, these observations provide a biological rationale for exploring whether blood-derived ex vivo cytokine-response features vary according to distant metastasis status and whether they are related to local immune-cell organization in the primary tumor-bearing mucosa (8).

However, the immune biology of CRC is not restricted to the tumor site. Circulating monocytes and blood-derived CD14+ cell populations may carry systemic signatures of tumor-associated immune reprogramming. Experimental and clinical studies suggest that peripheral monocytes in CRC can display altered phenotypic or functional properties, including changes that may influence macrophage differentiation and the composition of the tumor microenvironment (9). Nevertheless, systemic functional responsiveness of monocyte/macrophage-lineage cells remains less extensively characterized than tissue immune infiltration. This is particularly relevant because static measurements of cytokine concentration in blood, a cell culture or in a tissue provide only limited information about immune regulation. They do not capture how cells respond to inflammatory challenge, terminate or maintain cytokine secretion after stimulation, or react to repeated stimulation.

Dynamic ex vivo stimulation models may therefore provide additional information about the regulatory state of innate immune cells. Lipopolysaccharide (LPS) challenge is widely used to assess monocyte/macrophage inflammatory responsiveness, including the production of cytokines and chemokines such as IL-1β, IL-6, IL-8, MCP-1, and TNF-α. In a previous study, altered proinflammatory cytokine responses were observed in CD14+ monocyte cultures from patients with CRC, including impaired TNF-α response and enhanced IL-1β secretion after repeated LPS stimulation (10). These findings suggested that CRC may be associated with systemic innate immune dysregulation, but they did not resolve how broader cytokine-response dynamics relate to clinicopathological characteristics or to the local mucosal immune-cell context.

Unlike conventional immune phenotyping, which primarily characterizes cell abundance, marker expression, or mediator levels at individual time points, sequential stimulation enables functional response trajectories to be quantified across basal, activation, post-stimulation, and repeated-challenge states. The resulting dynamic indices may therefore provide complementary information on immune response regulation that is not captured by static measurements. In the present study, these indices were evaluated as research-level functional phenotypes; their potential clinical biomarker utility would require independent prospective validation.

IL-1β and IL-6 were selected as prototypic inflammatory cytokines with established relevance to CRC-associated inflammatory signaling. IL-1 signaling is involved in colon inflammation, carcinogenesis, tumor–stroma interactions, and inflammatory circuits that may promote or modulate tumor progression (11), whereas IL-6 is a central mediator of CRC-associated inflammation and has been linked to STAT3 activation, tumor-cell survival, proliferation, invasion, angiogenesis, immune modulation, and adverse clinical outcome (12). IL-8/CXCL8 and MCP-1/CCL2 were included as chemokines linking inflammatory activation to recruitment and functional modulation of myeloid and other immune-cell populations. Together, these analytes provided a focused panel spanning inflammatory cytokine production and myeloid-relevant chemokine output within the same sequential LPS stimulation model. The panel was not intended to provide comprehensive immune profiling. Other relevant mediators, including TNF-α and IL-10, could provide additional information on pro- and anti-inflammatory response programs, whereas cytokines such as IFN-γ and IL-17A predominantly represent immune axes not directly captured by the present CD14+ cell-derived culture model. Accordingly, the selected panel should be interpreted as a focused assessment of specific inflammatory response pathways rather than as a complete representation of CRC-associated immune regulation.

Another unresolved issue is how blood-derived ex vivo cytokine-response features relate to the local immune-cell composition of CRC-associated mucosa. Most spatial immune analyses in CRC have focused on tumor tissue and the invasive margin. At the same time, morphologically non-tumor or tumor-distant colorectal mucosa in CRC patients may not be an immunologically inert comparator. Prior studies have demonstrated immune differences between CRC tissue and normal mucosa, and have shown that immune-cell densities and immune-related gene expression in tumor-distant normal mucosa may vary according to cancer status and molecular background (13). More recent transcriptomic analyses also support the presence of field-like biological variability in non-tumor colorectal mucosa of CRC patients (14). These observations raise the possibility that tumor-adjacent mucosa and resection-margin mucosa may represent distinct but interconnected mucosal immune compartments.

In the present study, we investigated CRC-associated immune remodeling using complementary blood-derived ex vivo and tissue-level readouts. We analyzed dynamic cytokine-response indices in M-CSF-differentiated cultures derived from peripheral-blood CD14+ cells after sequential LPS stimulation and quantified CD4+, CD8+, CD20+, CD56+, and CD68+ cell densities in tumor-adjacent and resection-margin mucosa. We assessed CRC-associated differences in cytokine responsiveness, compartment-specific mucosal immune-cell organization, associations with histological grade, and relationships between cytokine-response indices and local immune-cell densities. As a secondary exploratory analysis, we also examined whether distant metastasis status was associated with additional differences in IL-1β, IL-6, IL-8, or MCP-1 response indices. Because only nine patients had distant metastases, this analysis was intended to identify preliminary metastasis-associated patterns rather than to establish predictive biomarkers or causal mechanisms of metastatic progression. We hypothesized that CRC is associated with selective alterations in cytokine responsiveness of CD14+ cell-derived cultures and that these ex vivo response features are partially related to local mucosal immune-cell composition.

## 2 Materials and methods

### 2.1 Study design and participants

This single-center prospective observational study was conducted at the Petrovsky National Research Centre of Surgery, Moscow, Russia, between May 2025 and December 2025. Eligible participants were recruited consecutively during the study period. The colorectal cancer (CRC) group included patients with histologically confirmed adenocarcinoma of the colon or rectum who underwent surgical treatment followed by routine pathological assessment of the resected specimens.

The comparison group included patients undergoing surgery for non-malignant, non-incarcerated abdominal wall hernias. Among comparison subjects, 8 men had non-incarcerated inguinal hernia, 3 men had non-incarcerated umbilical hernia, 6 women had non-incarcerated umbilical hernia, and 3 women had non-incarcerated inguinal hernia. The comparison group was selected to provide a non-malignant clinical cohort recruited within the same surgical setting and assessed using the same preanalytical procedures as the CRC group. It was not intended to represent a population-based healthy reference group. Peripheral blood was collected before surgery in both groups; therefore, the measured cytokine-response patterns were not affected by the acute inflammatory response to the surgical procedure itself.

Exclusion criteria included age below 40 or above 79 years, previous antitumor treatment before sampling, malignant tumors of other localizations within the previous 5 years, autoimmune diseases, history of hepatitis B, hepatitis C, or HIV infection, psychiatric disorders, alcohol or drug abuse, decompensated renal or hepatic failure, and chronic heart failure class III–IV according to the New York Heart Association classification.

The study involving human participants was reviewed and approved by the Local Ethics Committee of the Federal State Budgetary Scientific Institution “Russian Scientific Center of Surgery named after Academician B.V. Petrovsky”, Moscow, Russia, which serves as the institution’s authorized ethics review committee for human participant research. Ethical approval was granted under protocol/ethical approval No. 5 dated 23 of May 2025. Written informed consent was obtained from each participant before inclusion in the study. The study was conducted in accordance with the Declaration of Helsinki.

### 2.2 Clinical and pathological assessment

Clinical and pathological data were collected from medical records and pathology reports. For CRC patients, the analyzed variables included age, sex, primary tumor site, tumor sidedness, histological grade, AJCC/UICC stage, distant metastasis status, and KRAS/NRAS/BRAF mutation status. For tumor-sidedness analyses, tumors located proximal to the splenic flexure were classified as right-sided, whereas tumors of the splenic flexure and more distal colorectal segments, including the rectum, were classified as left-sided. Patients with multiple primary colorectal cancers were excluded from the tumor-sidedness analysis. Surgical specimens were subjected to routine pathological examination, including assessment of tumor histological type, grade of differentiation, depth of invasion, regional lymph node status, and distant metastasis status when applicable. Tumor stage was assigned according to the AJCC/UICC TNM classification. Primary tumor localization was classified as colon or rectum. Multiple primary colorectal cancers were recorded separately, in the present cohort, all multiple primary tumors were located within the colon.

### 2.3 Histology and immunohistochemistry

Surgical specimens were fixed in 10% neutral buffered formalin, processed using a Leica ASP 300 automated tissue processor (Leica Microsystems, Germany), and embedded in paraffin using a Leica EG 1150 embedding station (Leica Microsystems, Germany). Sections 4 μm thick were prepared from formalin-fixed paraffin-embedded tissue blocks and stained with hematoxylin and eosin using a Leica ST 5010 automated staining system (Leica Microsystems, Germany). Microscopic examination was performed using a Leica DMLB microscope equipped with a Leica DFC 420 digital camera (Leica Microsystems, Germany).

Immunohistochemistry was performed on automated staining platforms Leica Bond-maX (Leica Microsystems, Germany) and Roche Ventana BenchMark Ultra (Roche Diagnostics, USA). The following primary antibodies were used: CONFIRM™ anti-CD4 (SP35) Rabbit Monoclonal Primary Antibody, ready-to-use (Roche/Ventana); CONFIRM™ anti-CD8 (SP57) Rabbit Monoclonal Primary Antibody, ready-to-use (Roche/Ventana); CONFIRM™ anti-CD20 (L26) Primary Antibody, ready-to-use (Roche/Ventana); CD56 (MRQ-42) Rabbit Monoclonal Antibody, ready-to-use (Cell Marque); and CONFIRM™ anti-CD68 (KP-1) Primary Antibody, ready-to-use (Roche/Ventana).

Immunopositive cells were counted in the lamina propria mucosae in two compartments: tumor-adjacent mucosa and resection-margin mucosa. Tumor-adjacent mucosa was defined as mucosa immediately adjacent to the tumor. Resection-margin mucosa was defined as morphologically tumor-free mucosa from the surgical resection margin, located at least 6 cm from the tumor edge in colon cancer and at least 2 cm from the tumor edge in rectal cancer. Absence of tumor tissue in the resection margin was confirmed histologically.

Cell counting was performed by one pathologist in 10 randomly selected high-power fields at ×400 magnification for each marker and compartment. Fields adjacent to lymphoid follicles, prominent lymphoid aggregates were excluded. For each case, the mean number of immunopositive cells per visual field was calculated separately for each marker and tissue compartment.

### 2.4 CD14+ cell isolation

Peripheral venous blood was collected before surgery into vacuum tubes containing EDTA. For each participant, 30 mL of blood was used for CD14+ cell isolation. Peripheral blood mononuclear cells were first isolated by density-gradient centrifugation using Ficoll. CD14+ cells were then enriched by positive magnetic separation using CD14 MicroBeads, LS columns, and a QuadroMACS Separator system (Miltenyi Biotec, USA) according to the manufacturer’s principles.

After magnetic separation, viable cells were counted using a Goryaev chamber after trypan blue staining. Dead cells were excluded from the count. Only cell preparations with viability greater than 90% were used for subsequent culture.

### 2.5 M-CSF differentiation of CD14+ cells and sequential LPS stimulation

Isolated peripheral-blood CD14+ cells were cultured in multiwell plates at a density of 1 × 10^6^ viable cells/mL in RPMI-1640 medium (PanEco, Russia) supplemented with 10% fetal bovine serum (BioloT, Russia), 2 mM L-glutamine (PanEco, Russia), penicillin/streptomycin (PanEco, Russia), and macrophage colony-stimulating factor (M-CSF; CLOUD-CLONE CORP., CCC, USA) at a final concentration of 50 ng/mL. Cells were incubated at 37 °C in a humidified atmosphere containing 5% CO_2_, and the medium was replaced on day 3 using the same culture conditions.

The five-day M-CSF-driven differentiation period was used to generate CD14+ cell-derived cultures under macrophage-differentiating conditions before assessment of cytokine responsiveness. Accordingly, the experimental readouts reflect cytokine-response properties retained or acquired after ex vivo M-CSF-driven differentiation and should not be interpreted as direct measurements of freshly isolated circulating monocytes. Because post-differentiation cellular phenotype was not characterized using additional surface or transcriptional markers, the cultures are referred to throughout the manuscript as M-CSF-differentiated cultures derived from peripheral-blood CD14+ cells rather than as fully characterized macrophages.

On day 6, the culture medium was removed, cells were washed with phosphate-buffered saline, and fresh culture medium without M-CSF was added. Three wells were used for each participant. The first well was maintained under basal conditions without LPS stimulation. The second well was stimulated once with LPS at a final concentration of 100 ng/mL (Sigma, USA). The third well was also stimulated with LPS at 100 ng/mL and was subsequently used for repeated LPS stimulation.

After 24 h, culture supernatants were collected. Basal secretion after 24 h was measured from the unstimulated well and designated B24. The primary LPS response was assessed from the two LPS-stimulated wells after the first 24 h of stimulation, the mean value of these two wells was designated LPS1. After supernatant collection, cells were washed with phosphate-buffered saline and fresh medium was added. The unstimulated well was maintained for an additional 24 h without LPS to assess basal secretion after 48 h, designated B48. The second well was maintained for 24 h without repeated LPS stimulation to assess post-stimulation persistence after LPS washout, designated PERSIST. The third well was stimulated again with LPS at 100 ng/mL for 24 h to assess the response to repeated LPS stimulation, designated LPS2.

### 2.6 Cytokine measurement

Concentrations of IL-1β, IL-6, IL-8, and MCP-1 in culture supernatants were measured by enzyme-linked immunosorbent assay using commercial DuoSet ELISA kits: Human IL-1β/IL-1F2, Human IL-6, Human IL-8/CXCL8, and Human CCL2/MCP-1 (R&D Systems, USA). Assays were performed according to the manufacturer’s instructions.

Optical density was measured using a Multiskan FC microplate photometer with an integrated incubator (Thermo Fisher Scientific, USA). Absorbance was read at 450 nm with wavelength correction at 570 nm, according to the kit recommendations. Samples were measured in duplicate. Standard curves were generated using serial dilutions of the corresponding recombinant standards and fitted using a four-parameter logistic model. Values below the detection range were assigned a value of zero. Samples exceeding the calibration range were diluted, remeasured, and corrected for the dilution factor.

Because cultures were established at 1 × 10^6^ cells/mL, cytokine concentrations were expressed as pg/mL per 10^6^ cells before calculation of dynamic indices.

### 2.7 Dynamic cytokine-response indices

Dynamic cytokine-response indices were calculated separately for each cytokine using cytokine concentrations measured at five experimental points: B24, B48, LPS1, PERSIST, and LPS2. Before index calculation, cytokine concentrations were transformed using the log1p transformation.

The following indices were calculated (**Table 1**):

**Table 1:** Cytokine dynamic index calculation.

Dynamic index	Calculation
Basal activity	B24
Basal drift	B48 − B24
Primary LPS response	LPS1 − B24
Persistence	PERSIST − B48
Shutdown efficiency	LPS1 − PERSIST
Re-induction capacity	LPS2 − PERSIST
Re-stimulation index	LPS2 − LPS1

Basal activity reflected basal cytokine secretion after 24 h. Basal drift reflected the change in basal secretion between 24 and 48 h in the absence of LPS stimulation. Primary LPS response reflected the increase in cytokine secretion after the first LPS challenge relative to basal secretion. Persistence reflected residual post-stimulation cytokine secretion after LPS washout. Shutdown efficiency reflected the decrease in cytokine secretion after removal of the first LPS stimulus. Re-induction capacity reflected the response to repeated LPS stimulation relative to the post-washout state. The re-stimulation index reflected the difference between the second and first LPS-induced responses.

### 2.8 DNA extraction and KRAS/NRAS/BRAF mutation testing

*KRAS*, *NRAS*, and *BRAF* mutation status was assessed using DNA extracted from FFPE tumor tissue. Before DNA extraction, tumor material was reviewed to ensure that tumor content met the requirements specified by the mutation testing kit manufacturer.

DNA was extracted using the DNA-Tissue-M kit (TestGen, Russia) according to the manufacturer’s instructions. The procedure included deparaffinization, proteinase K digestion, magnetic-particle-based DNA purification, and elution. DNA concentration and purity were assessed spectrophotometrically; samples with A260/A280 ratio ≥1.7 were considered suitable for subsequent analysis.

Mutation testing was performed by real-time PCR using validated commercial kits for KRAS, NRAS, and BRAF mutation detection: Test-KRAS-tissue, Test-NRAS-tissue, and Test-BRAF-tissue-multi (TestGen, Russia), according to the manufacturer’s instructions. Amplification and detection were performed on a CFX96 real-time PCR system (Bio-Rad, USA). The assays included an endogenous internal control for human DNA quality and amplifiability. All analyzed samples met the manufacturer’s quality-control criteria.

### 2.9 Statistical analysis

Statistical analysis was performed using Python with the pandas, NumPy, SciPy, statsmodels, and matplotlib libraries. Continuous variables were summarized as mean ± standard deviation or median with interquartile range [IQR], as appropriate. Categorical variables were summarized as absolute counts and percentages.

Cytokine concentrations were transformed using the log1p transformation before calculation of dynamic cytokine-response indices. Between-group comparisons of dynamic cytokine-response indices were performed using nonparametric tests. For comparisons between two independent groups, the Mann–Whitney U test was used. For comparisons across more than two groups, the Kruskal–Wallis test was used where applicable. For the main comparison between CRC patients and the comparison group, selected dynamic indices were additionally analyzed using age- and sex-adjusted robust linear regression models. In these models, the dynamic cytokine-response index was used as the dependent variable, study group was used as the main predictor, and age and sex were included as covariates. Adjusted β coefficients and 95% confidence intervals were reported on the log1p-transformed index scale.

For the analysis of distant metastasis status within the CRC cohort, dynamic cytokine-response indices were compared between M0 and M1 patients. Age- and sex-adjusted robust linear regression models were used to estimate the association between M1 status and each cytokine-response index. In these models, the dynamic index was used as the dependent variable, distant metastasis status was used as the main predictor, and age and sex were included as covariates. Positive β values indicated higher index values in M1 patients, whereas negative β values indicated lower index values in M1 patients.

No statistical imputation of missing data was performed. Analyses were based on available observations. Paired analyses used complete cases for the corresponding variables, and correlation analyses used pairwise complete observations.

Paired comparisons of immunohistochemical immune-cell densities between tumor-adjacent mucosa and resection-margin mucosa were performed using complete cases for each marker. Normality of paired differences was assessed using the Shapiro–Wilk test. If the distribution of differences was compatible with normality, the paired t-test was used; otherwise, the Wilcoxon signed-rank test was applied. Immune-cell densities were summarized as median [IQR] and expressed as cells per visual field at ×400 magnification.

Correlation analyses were performed to assess associations between local immune-cell densities, clinicopathological variables, and dynamic cytokine-response indices. Pairwise complete observations were used for each correlation. Spearman rank correlation was used for associations involving quantitative non-normally distributed variables or ordinal variables. For ordinal–ordinal associations, Kendall’s tau-b was used where applicable. Correlation coefficients were reported with 95% confidence intervals estimated using percentile bootstrap with 5000 iterations. Scatter plots were used for selected representative correlations, and trend lines were shown for visualization only.

For heatmap-based systemic–local analyses, Spearman correlation coefficients were calculated for each pair of selected dynamic cytokine-response indices and local immune-cell density variables. The corresponding p values were adjusted for multiple testing across all successfully calculated heatmap cells. Heatmap cell color represented Spearman ρ, and statistically significant associations after FDR correction were marked graphically.

Exploratory mutation-specific analyses were performed separately for patients with KRAS-, NRAS-, and BRAF-mutated tumors, using patients with wild-type status for all three genes as the reference group. Dynamic cytokine-response indices and local immune-cell densities were compared using the Mann–Whitney U test.

Multiple testing was controlled using the Benjamini–Hochberg false discovery rate (FDR) procedure. For the mutation-specific analyses, FDR correction was applied separately to the cytokine-response and immunohistochemical analysis families. FDR correction was applied within predefined analysis families corresponding to the main analytical blocks: CRC-versus-comparison cytokine-response analyses, metastasis-associated cytokine-response analyses, paired immunohistochemical comparisons, local immune-cell correlation analyses, and systemic–local correlation analyses. For the predefined matched-marker analysis between tumor-adjacent and resection-margin mucosa, FDR correction was applied across the five matched immune-cell marker correlations. Results were considered statistically significant at FDR q < 0.05.

## 3 Results

### 3.1 Cohort characteristics and study workflow

A total of 126 participants were included in the study, including 106 patients with colorectal cancer (CRC) and 20 comparison subjects with non-malignant surgical conditions. No formal *a priori* sample-size or power calculation was performed. The study size was determined by the number of eligible participants enrolled during the predefined recruitment period. The CRC and comparison groups were comparable in age and sex distribution: mean age was 70.9 ± 8.2 years in CRC patients and 71.1 ± 8.1 years in comparison subjects. Males accounted for 56.6% and 55.0% of the two groups, respectively. Peripheral blood sampling and CD14+ cell culture were performed for all participants. None of the CRC patients had received antitumor therapy before sampling.

Among CRC patients, primary tumor localization was available for all 106 cases: 74 patients had colon cancer and 32 had rectal cancer. Five patients had multiple primary colorectal cancers, all located within the colon. Patients with multiple primary colorectal cancers were excluded from the tumor-sidedness analysis. According to AJCC/UICC staging, 38 patients had stage I disease, 27 had stage II, 32 had stage III, and 9 had stage IV disease (distant metastases were present in 9 patients). Histological grade was distributed as G1 in 25 patients, G2 in 66 patients, and G3 in 15 patients. KRAS/NRAS/BRAF mutations were detected in 46 patients, whereas 60 patients had wild-type status for these genes (**Table 2**).

**Table 2:** Study cohort characteristics.

Characteristic	CRC patients, n = 106	Comparison group, n = 20	p value
Demographic characteristics			
Age, years, mean ± SD	70.9 ± 8.2	71.1 ± 8.1	0.938
Sex, n (%)			
Male	60 (56.6)	11 (55.0)	0.894
Female	46 (43.4)	9 (45.0)	
Study group characteristics			
Core clinicopathological characteristics of CRC patients			
Primary tumor site, n/N (%)		Not applicable	—
Colon	74/106 (69.8)	Not applicable	—
Rectum	32/106 (30.2)	Not applicable	—
Multiple primary colorectal cancer, n (%)	5/106 (4.7)	Not applicable	—
Tumor sidedness, n (%)		Not applicable	—
Right-sided	26/101 (25.7)	Not applicable	—
Left-sided	75/101 (74.3)	Not applicable	—
Not classifiable/excluded from sidedness analysis	5/106 (4.7)	Not applicable	
AJCC/UICC stage, n (%)		Not applicable	—
I	38 (35.8)	Not applicable	—
II	27 (25.5)	Not applicable	—
III	32 (30.2)	Not applicable	—
IV	9 (8.5)	Not applicable	—
Distant metastasis, n (%)		Not applicable	—
M0	97 (91.5)	Not applicable	—
M1	9 (8.5)	Not applicable	—
Histological grade, n (%)		Not applicable	—
G1	25 (23.6)	Not applicable	—
G2	66 (62.3)	Not applicable	—
G3	15 (14.1)	Not applicable	—
KRAS/NRAS/BRAF status, n (%)		Not applicable	—
Wild type	60 (56.6)	Not applicable	—
Mutated	46 (43.4)	Not applicable	—

**Figure 1:**
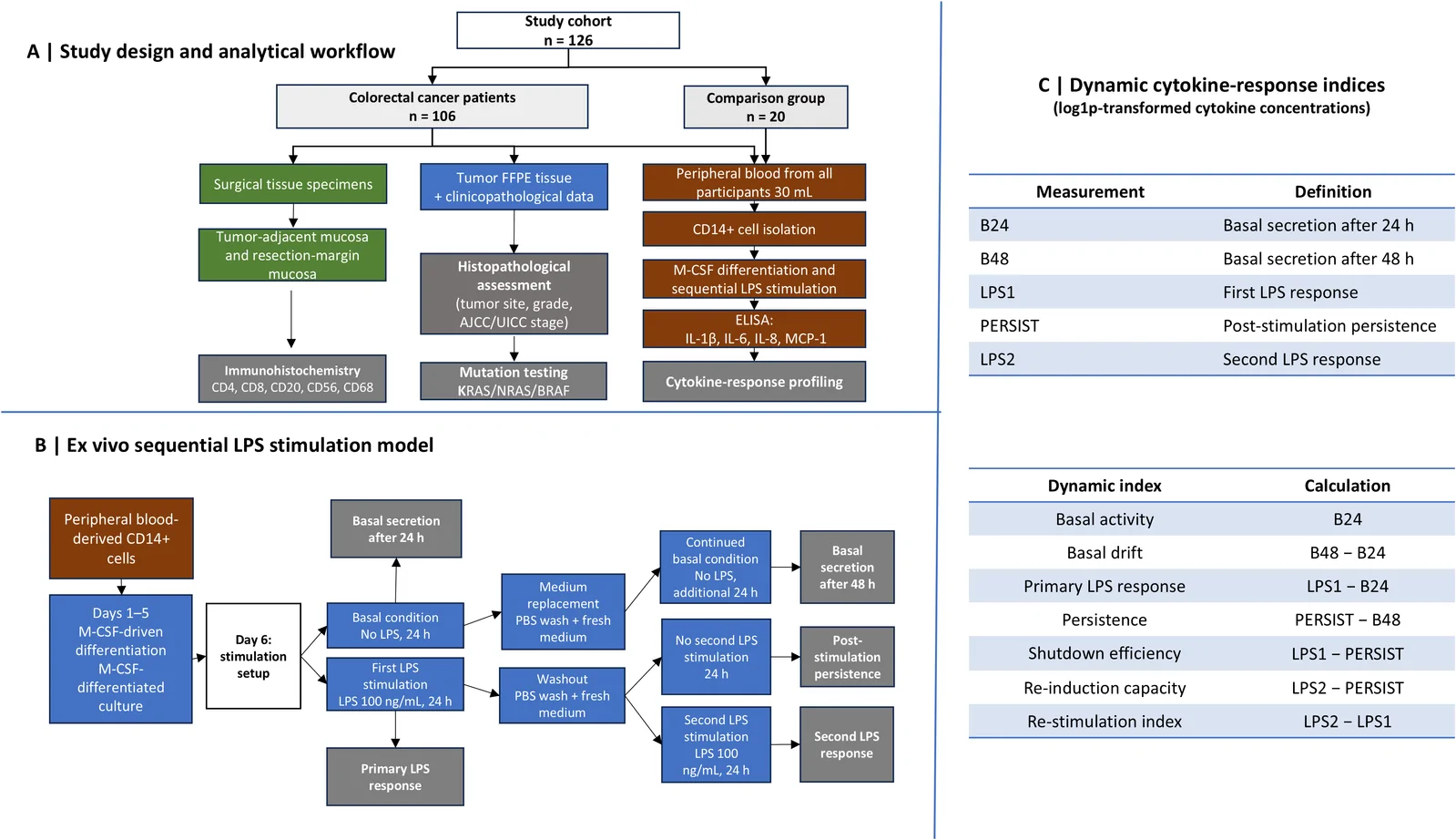
Study design, M-CSF differentiation and ex vivo sequential LPS stimulation model, and cytokine-response indices. **(A)** Overview of the study design and analytical workflow. The study cohort included 106 patients with colorectal cancer and 20 comparison subjects. Peripheral blood was obtained from all participants for CD14+ cell isolation, M-CSF-driven differentiation, sequential LPS stimulation, and cytokine-response profiling. In patients with colorectal cancer, surgical tissue specimens were additionally used for immunohistochemical assessment of CD4+, CD8+, CD20+, CD56+, and CD68+ cells in tumor-adjacent and resection-margin mucosa. Tumor FFPE tissue and clinicopathological data were used for histopathological assessment, AJCC/UICC staging, and KRAS/NRAS/BRAF mutation testing. **(B)** Ex vivo model of M-CSF differentiation and sequential LPS stimulation. Peripheral-blood CD14+ cells were cultured under M-CSF-driven macrophage-differentiating conditions for five days, with sequential LPS stimulation initiated on day 6. Cytokine secretion by the resulting M-CSF-differentiated cultures was assessed under basal conditions, after the first LPS stimulation, after medium replacement and LPS washout, and after the second LPS stimulation. Culture supernatants were collected for cytokine measurement at the indicated experimental points. This model assesses cytokine responsiveness after ex vivo differentiation and does not represent a direct functional assay of freshly isolated circulating monocytes. **(C)** Cytokine-response indices calculated from log1p-transformed cytokine concentrations. B24 and B48 denote basal cytokine secretion after 24 and 48 h, respectively; LPS1 denotes the cytokine response after the first LPS stimulation; PERSIST denotes post-stimulation persistence after LPS washout without repeated stimulation; and LPS2 denotes the cytokine response after the second LPS stimulation. AJCC, American Joint Committee on Cancer; CRC, colorectal cancer; ELISA, enzyme-linked immunosorbent assay; FFPE, formalin-fixed paraffin-embedded; LPS, lipopolysaccharide; M-CSF, macrophage colony-stimulating factor; UICC, Union for International Cancer Control.

The study design, analytical workflow, sequential LPS stimulation model, and dynamic cytokine-response indices are summarized in **Figure 1**.

### 3.2 CRC is associated with altered dynamic cytokine responsiveness

**Figure 2:**
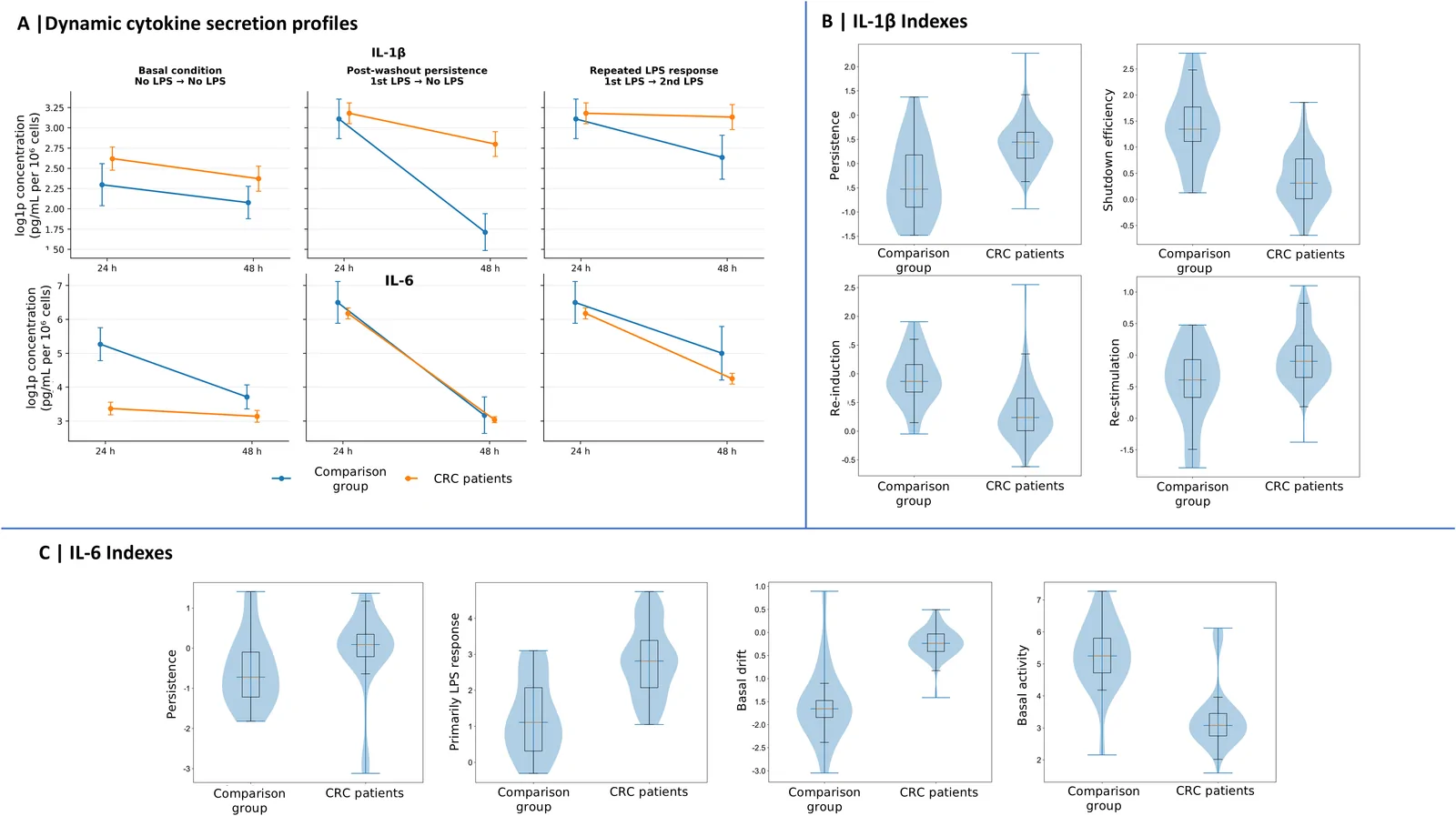
Altered dynamic IL-1β and IL-6 secretion profiles in colorectal cancer. **(A)** Dynamic secretion profiles of IL-1β and IL-6 in the comparison group and in patients with colorectal cancer (CRC). Slope plots show three experimentally defined two-point trajectories: basal condition (No LPS → No LPS), post-washout persistence after the first LPS challenge (1st LPS → No LPS), and repeated LPS response (1st LPS → 2nd LPS). Cytokine concentrations were analyzed after log1p transformation. Points indicate group means; error bars indicate 95% confidence intervals. **(B)** Distribution of selected IL-1β dynamic response indices in the comparison group and CRC patients. **(C)** Distribution of selected IL-6 dynamic response indices in the comparison group and CRC patients. Violin plots show the distribution of individual index values, with embedded boxplots indicating the median and interquartile range. CRC, colorectal cancer; LPS, lipopolysaccharide.

Dynamic cytokine-response profiling revealed distinct IL-1β and IL-6 secretion patterns in CRC patients compared with the comparison group (**Figure 2**).

For IL-1β, the most prominent differences were observed after the first LPS stimulation and during the subsequent post-washout and re-stimulation phases. CRC patients showed higher post-washout persistence of IL-1β secretion than comparison subjects, together with markedly lower shutdown efficiency and reduced re-induction capacity. The re-stimulation index was also higher in CRC patients, indicating a relative preservation of the second LPS-induced response compared with the primary LPS response.

These patterns were supported by quantitative comparison of dynamic indices (**Table 3**). IL-1β persistence was higher in CRC patients than in comparison subjects (median 0.44 [0.11; 0.65] vs −0.53 [−0.90; 0.18], FDR q < 0.001), whereas shutdown efficiency and re-induction capacity were lower in CRC patients (both FDR q < 0.001). The IL-1β re-stimulation index also differed between groups (FDR q = 0.023). These differences remained significant in age- and sex-adjusted robust linear models, with adjusted β values consistent with higher persistence and re-stimulation index but lower shutdown efficiency and re-induction capacity in CRC patients.

**Table 3:** Differences in dynamic IL-1β and IL-6 response indices between CRC patients and comparison subjects.

Cytokine	Dynamic index	Comparison group, median [IQR]	CRC patients, median [IQR]	p value	FDR q value	Age- and sex-adjusted β [95% CI], log1p scale	Adjusted FDR q value
IL-1β	Persistence	−0.53 [−0.90; 0.18]	0.44 [0.11; 0.65]	<0.001	<0.001	0.83 [0.60; 1.07]	<0.001
IL-1β	Shutdown efficiency	1.34 [1.11; 1.77]	0.31 [0.01; 0.77]	<0.001	<0.001	−1.02 [−1.28; −0.75]	<0.001
IL-1β	Re-induction capacity	0.86 [0.68; 1.16]	0.24 [0.01; 0.57]	<0.001	<0.001	−0.63 [−0.83; −0.42]	<0.001
IL-1β	Re-stimulation index	−0.39 [−0.67; −0.07]	−0.10 [−0.35; 0.15]	0.007	0.023	0.30 [0.09; 0.50]	0.016
IL-6	Basal activity	5.25 [4.72; 5.80]	3.07 [2.75; 3.45]	<0.001	<0.001	−2.13 [−2.41; −1.84]	<0.001
IL-6	Basal drift	−1.66 [−1.84; −1.48]	−0.24 [−0.41; −0.03]	<0.001	<0.001	1.44 [1.30; 1.58]	<0.001
IL-6	Primary LPS response	1.11 [0.31; 2.07]	2.81 [2.07; 3.38]	<0.001	<0.001	1.59 [1.05; 2.12]	<0.001
IL-6	Persistence	−0.72 [−1.22; −0.10]	0.09 [−0.21; 0.35]	0.004	0.015	0.75 [0.48; 1.01]	<0.001

Dynamic cytokine-response indices were calculated from log1p-transformed cytokine concentrations. Values are presented as median [interquartile range]. Between-group comparisons were performed using the Mann–Whitney U test. q values represent FDR-adjusted p values. Adjusted β coefficients were estimated using robust linear regression models adjusted for age and sex and are expressed on the log1p-transformed index scale. Positive β values indicate higher index values in CRC patients, whereas negative β values indicate lower index values in CRC patients. CRC, colorectal cancer; CI, confidence interval; FDR, false discovery rate; IQR, interquartile range; LPS, lipopolysaccharide.

IL-6 dynamics showed a distinct pattern. CRC patients had lower basal IL-6 activity at 24 h than comparison subjects, but demonstrated higher basal drift, stronger primary LPS response, and higher post-washout persistence. IL-6 basal activity was lower in CRC patients (3.07 [2.75; 3.45] vs 5.25 [4.72; 5.80], FDR q < 0.001), whereas basal drift, primary LPS response, and persistence were higher in CRC patients, with all differences remaining significant after adjustment for age and sex (**Table 3**).

Together, these findings indicate that CRC is associated with selective remodeling of dynamic cytokine indices in M-CSF-differentiated cultures derived from peripheral-blood CD14+ cells, predominantly by altered IL-1β response persistence and resolution, and by a distinct shift in IL-6 basal and LPS-induced response dynamics. No FDR-significant between-group differences were observed for MCP-1 or IL-8 dynamic indices in the corresponding analyses. The full CRC-versus-comparison analysis across all cytokines and dynamic cytokine-response indices is provided in **Supplementary Table 1**. Distributions of IL-8 and MCP-1 dynamic response indices are shown in **Supplementary Figure 2**.

### 3.3 Exploratory analysis of cytokine-response patterns according to distant metastasis status

As a secondary exploratory analysis, cytokine-response indices were compared between 97 patients without distant metastases and 9 patients with distant metastases. Age- and sex-adjusted models were used to estimate the direction and magnitude of associations between distant metastasis status and each cytokine-response index. Given the small number of patients with distant metastases, this analysis was intended to identify preliminary patterns for subsequent validation rather than to assess predictive performance or infer mechanisms of metastatic dissemination.

**Figure 3:**
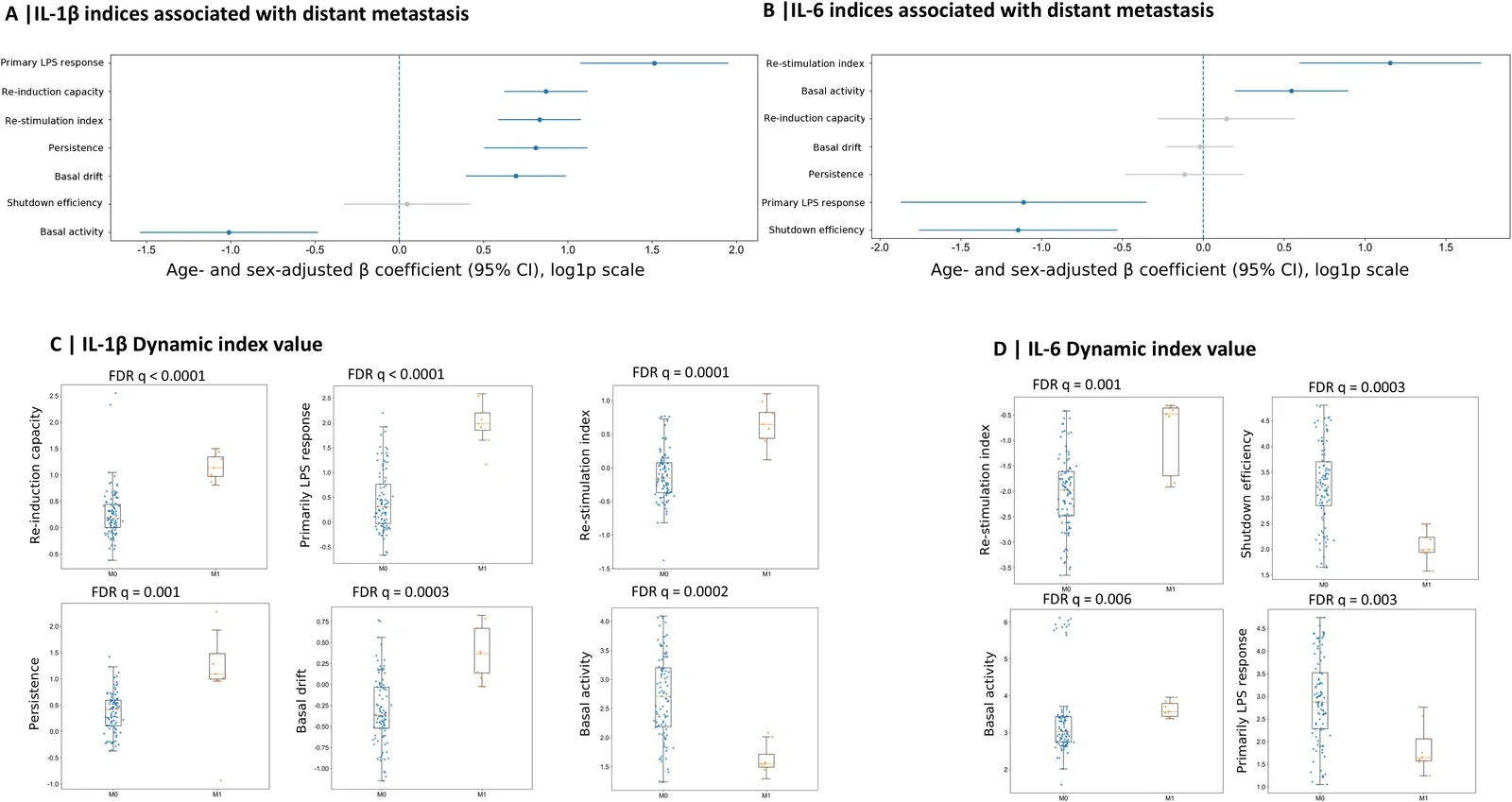
Exploratory associations between distant metastasis status and dynamic IL-1β and IL-6 response indices in colorectal cancer. **(A, B)** Age- and sex-adjusted associations between distant metastasis status and dynamic cytokine-response indices for IL-1β **(A)** and IL-6 **(B)**. Forest plots show adjusted β coefficients with 95% confidence intervals for M1 versus M0 status. Positive β values indicate higher index values in patients with distant metastases, whereas negative β values indicate lower index values in patients with distant metastases. Blue points and confidence intervals denote indices significant after FDR correction in the adjusted models (q < 0.05); gray points and confidence intervals denote non-significant indices. **(C, D)** Distributions of selected dynamic response indices that differed significantly between patients without and with distant metastases in unadjusted comparisons for IL-1β **(C)** and IL-6 **(D)**. Boxplots show the median and interquartile range, with individual points representing individual patients. q values indicate FDR-adjusted p values for between-group comparisons. All indices were calculated from log1p-transformed cytokine concentrations. This secondary analysis included 97 patients with M0 disease and 9 patients with M1 disease and should be interpreted as exploratory. CRC, colorectal cancer; CI, confidence interval; FDR, false discovery rate; IL, interleukin; LPS, lipopolysaccharide; M0, absence of distant metastases; M1, presence of distant metastases.

For IL-1β, differences according to distant metastasis status were observed across several response indices (**Figures 3A, C**). In age- and sex-adjusted models, the presence of distant metastases was associated with lower basal activity and higher basal drift, primary LPS response, persistence, re-induction capacity, and re-stimulation index. Shutdown efficiency did not remain significant after FDR correction in the adjusted model. Consistently, unadjusted comparisons showed higher primary LPS response, persistence, re-induction capacity, re-stimulation index, and basal drift in patients with distant metastases, together with lower basal activity.

IL-6 showed a different pattern of associations (**Figures 3B, D**). In adjusted models, the presence of distant metastases was associated with higher basal activity and a higher re-stimulation index, whereas primary LPS response and shutdown efficiency were lower in patients with distant metastases. Basal drift, persistence, and re-induction capacity did not remain significant after FDR correction in the adjusted models. The corresponding distribution plots also showed differences in IL-6 basal activity, primary LPS response, shutdown efficiency, and re-stimulation index between patients without and with distant metastases.

MCP-1 and IL-8 did not show comparable FDR-significant associations with distant metastasis status in the adjusted analyses. Overall, this exploratory comparison identified differences in selected IL-1β and IL-6 response indices between patients without and with distant metastases. These preliminary metastasis-associated patterns were observed in M-CSF-differentiated cultures derived from blood CD14+ cells and require confirmation in larger cohorts with adequate representation of metastatic disease. The full clinicopathological analysis of cytokine-response indices in CRC patients, including all cytokines, indices, and analyzed clinical variables, is provided in **Supplementary Table 2**.

Additional exploratory analyses were performed according to tumor mutation status. The CRC cohort included 29 patients with KRAS-mutated tumors, 9 with NRAS-mutated tumors, 8 with BRAF-mutated tumors, and 60 patients with wild-type status for all three genes. When each mutation-specific subgroup was compared with the triple-wild-type group, no differences in dynamic cytokine-response indices remained significant after FDR correction. Similarly, no mutation-specific differences in tumor-adjacent or resection-margin immune-cell densities remained significant after FDR correction. Full results are provided in **Supplementary Table 6**.

### 3.4 Local mucosal immune remodeling in CRC

**Figure 4:**
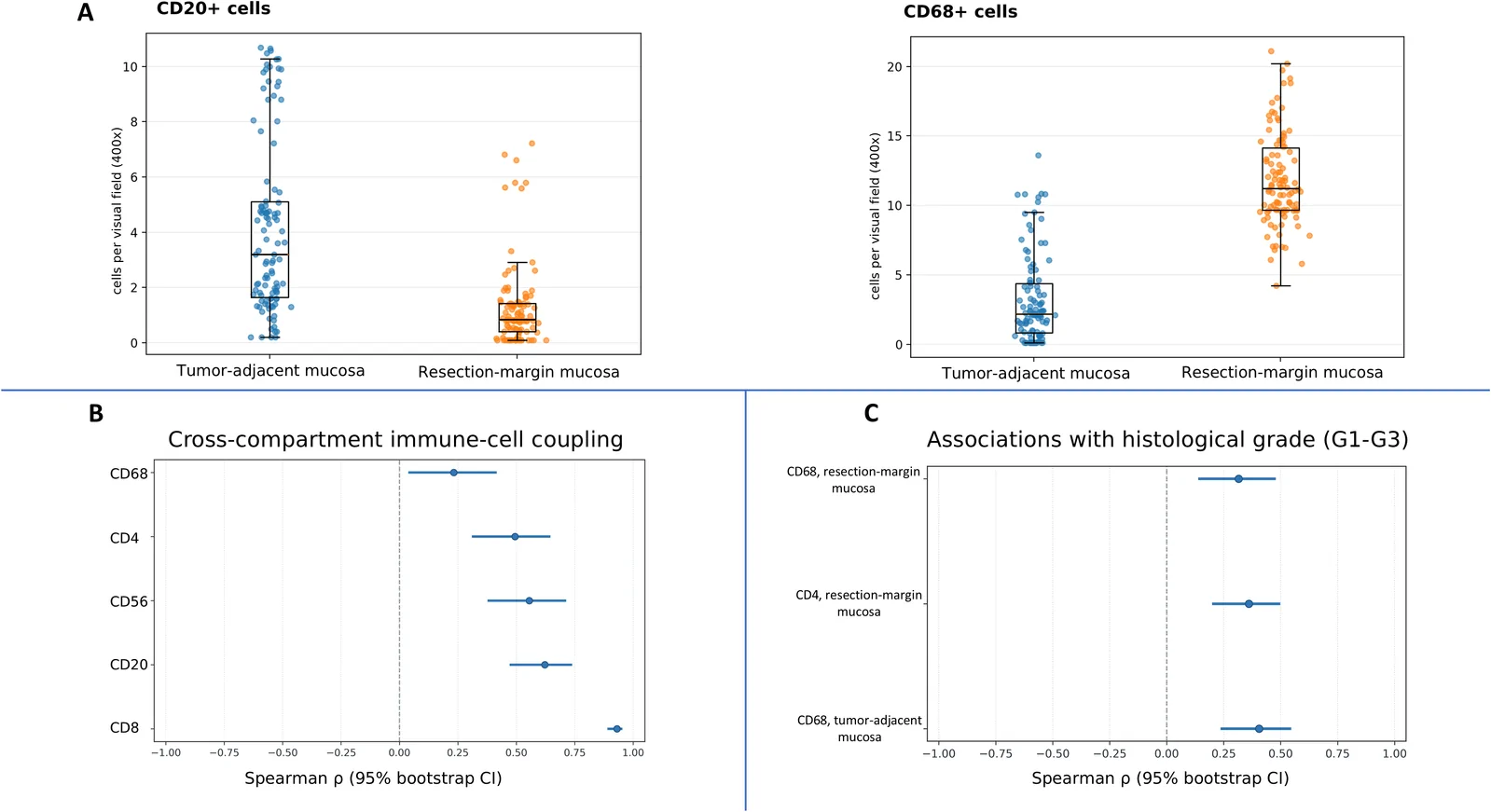
Local mucosal immune remodeling in colorectal cancer. **(A)** Compartment-specific differences in immune-cell densities between tumor-adjacent mucosa and resection-margin mucosa. Boxplots with individual data points show CD20+ and CD68+ cell densities, expressed as cells per visual field at ×400 magnification. CD20+ cells were enriched in tumor-adjacent mucosa, whereas CD68+ cells were enriched in resection-margin mucosa. **(B)** Cross-compartment immune-cell coupling between tumor-adjacent and resection-margin mucosa. Forest plots show Spearman correlation coefficients (ρ) with 95% bootstrap confidence intervals for matched immune-cell markers measured in both tissue compartments. **(C)** Associations between local immune-cell densities and histological grade. Forest plots show Spearman correlation coefficients (ρ) with 95% bootstrap confidence intervals for grade-associated immune-cell markers. Boxplots show the median and interquartile range; points represent individual patients. In correlation plots, points indicate Spearman ρ and horizontal lines indicate 95% confidence intervals estimated by percentile bootstrap. Only comparisons and correlations that remained significant after FDR correction are shown (q < 0.05). For panel **(B)**, FDR correction was applied within the predefined family of matched tumor-adjacent versus resection-margin marker correlations. CRC, colorectal cancer; CI, confidence interval; FDR, false discovery rate; ρ, Spearman correlation coefficient.

Immunohistochemical analysis revealed compartment-specific differences in local immune-cell composition between tumor-adjacent mucosa and resection-margin mucosa. Among the analyzed markers, significant paired differences were observed for CD20+ and CD68+ cells. CD20+ cell density was higher in tumor-adjacent mucosa than in resection-margin mucosa (median 3.20 [1.64; 5.12] vs 0.82 [0.40; 1.43] cells per visual field, FDR q = 4.50 × 10^-18^). In contrast, CD68+ cell density was markedly higher in resection-margin mucosa than in tumor-adjacent mucosa (11.24 [9.65; 14.16] vs 2.20 [0.82; 4.37] cells per visual field, FDR q = 1.99 × 10^-18^) (**Figure 4A**). No significant compartment-specific differences were observed for CD4+, CD8+, or CD56+ cell densities after FDR correction.

Despite these marker-specific compartmental differences, matched immune-cell densities showed coordinated variation between tumor-adjacent and resection-margin mucosa (**Figure 4B**). Cross-compartment correlations were strongest for CD8+ cells (Spearman ρ = 0.931, 95% bootstrap CI 0.892–0.955), indicating highly consistent interindividual ranking of CD8+ cell density across the two tissue compartments. Positive matched-marker correlations were also observed for CD20+ cells (ρ = 0.622, 95% CI 0.480–0.737), CD56+ cells (ρ = 0.555, 95% CI 0.370–0.710), CD4+ cells (ρ = 0.495, 95% CI 0.312–0.645), and CD68+ cells (ρ = 0.232, 95% CI 0.034–0.406). All five matched-marker correlations remained significant after FDR correction within the predefined cross-compartment correlation family.

Local immune-cell densities were also associated with histological grade (G1-G3) (**Figure 4C**). Higher histological grade was positively correlated with CD68+ cell density in tumor-adjacent mucosa (ρ = 0.405, 95% CI 0.236–0.546) and in resection-margin mucosa (ρ = 0.316, 95% CI 0.138–0.479). CD4+ cell density in resection-margin mucosa was also positively associated with grade (ρ = 0.361, 95% CI 0.199–0.499). These associations remained significant after FDR correction in the full local correlation analysis.

Together, these findings indicate that local mucosal immune remodeling in CRC involves both compartment-specific redistribution of selected immune-cell populations and coordinated immune-cell patterns across mucosal compartments, with macrophage- and CD4-associated markers linked to higher histological grade. Full paired immunohistochemical comparisons between tumor-adjacent and resection-margin mucosa are provided in **Supplementary Table 3**. The complete local immune-cell correlation analysis, including matched-marker cross-compartment correlations and associations with clinicopathological variables, is provided in **Supplementary Table 4**.

### 3.5 Ex vivo cytokine-response dynamics are associated with local immune-cell composition

**Figure 5:**
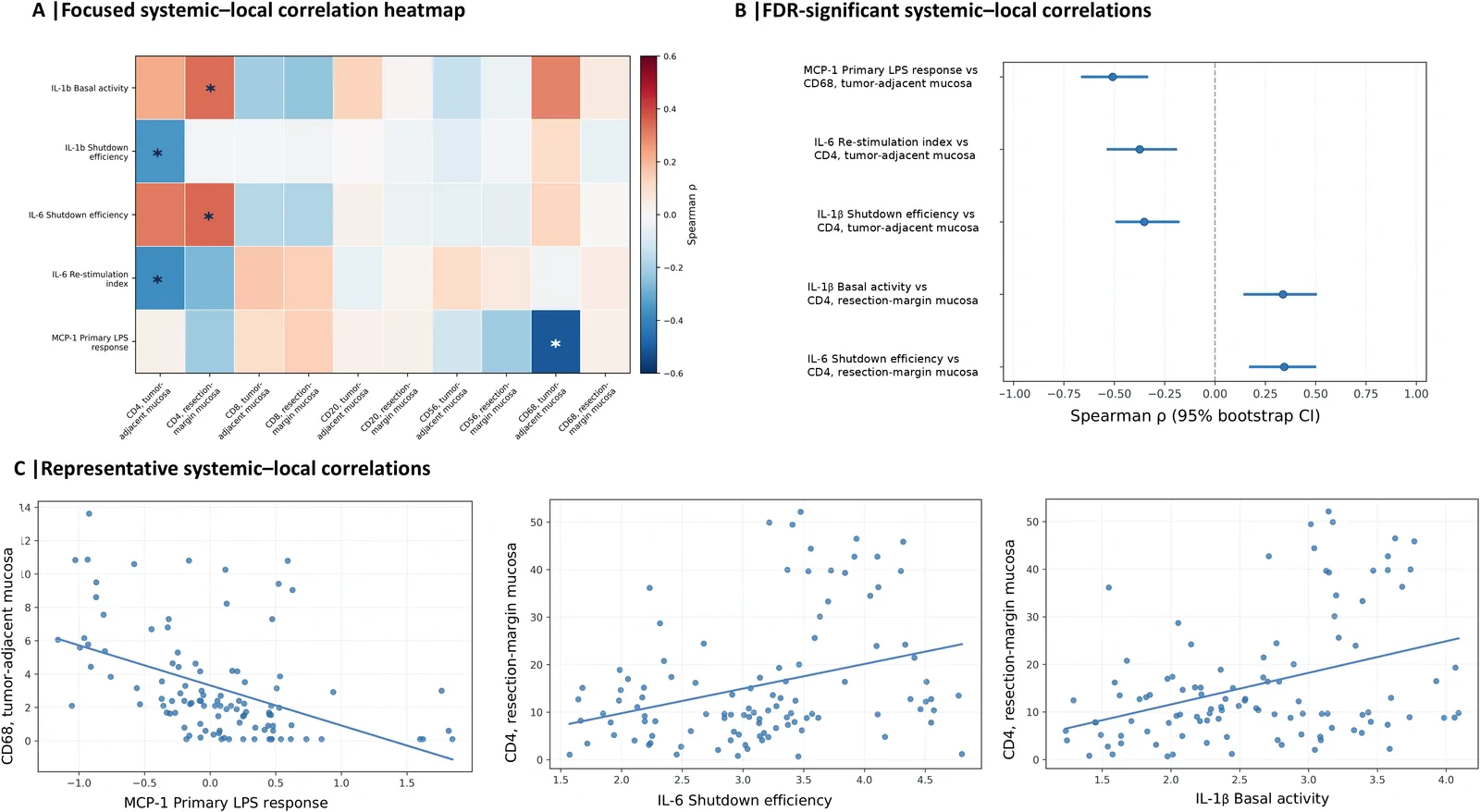
Associations between ex vivo cytokine-response dynamics and local immune-cell composition in colorectal cancer. **(A)** Focused heatmap of correlations between dynamic cytokine-response indices and local immune-cell densities. The heatmap includes cytokine-response indices with at least one FDR-significant association with local immune-cell markers. Cell color indicates Spearman correlation coefficient (ρ); asterisks indicate FDR-significant correlations (q < 0.05). **(B)** Forest plot of FDR-significant cross-domain correlations. Points indicate Spearman ρ, and horizontal lines indicate 95% confidence intervals estimated by percentile bootstrap. **(C)** Representative scatter plots illustrating selected systemic–local correlations: MCP-1 primary LPS response with CD68+ cell density in tumor-adjacent mucosa, IL-6 shutdown efficiency with CD4+ cell density in resection-margin mucosa, and IL-1β basal activity with CD4+ cell density in resection-margin mucosa. Trend lines are shown for visualization. All cytokine-response indices were calculated from log1p-transformed cytokine concentrations. Local immune-cell densities are expressed as cells per visual field at ×400 magnification. CI, confidence interval; FDR, false discovery rate; IL, interleukin; LPS, lipopolysaccharide; MCP-1, monocyte chemoattractant protein-1; ρ, Spearman correlation coefficient.

To determine whether cytokine-response dynamics measured in M-CSF-differentiated cultures derived from peripheral-blood CD14+ cells were related to local mucosal immune-cell composition, we analyzed correlations between cytokine-response indices and immunohistochemical immune-cell densities in tumor-adjacent and resection-margin mucosa. A focused correlation heatmap was constructed for cytokine-response indices with at least one FDR-significant association with local immune-cell markers (**Figure 5A**). The complete correlation heatmap, including all cytokine-response indices and local immune-cell variables, is shown in **Supplementary Figure 1**. Full numerical results for all correlations are provided in **Supplementary Table 5**.

Five cross-domain correlations remained significant after FDR correction (**Figure 5B**). The strongest association was observed between MCP-1 primary LPS response and CD68+ cell density in tumor-adjacent mucosa, showing an inverse correlation (Spearman ρ = −0.508, 95% bootstrap CI −0.665 to −0.332, FDR q = 7.42 × 10^-6^). Thus, higher CD68+ cell density in tumor-adjacent mucosa was associated with a lower MCP-1 response to primary LPS stimulation in the ex vivo CD14+ cell-derived culture model.

CD4+ cell density was linked to several IL-1β and IL-6 dynamic indices. In tumor-adjacent mucosa, CD4+ cell density correlated inversely with IL-6 re-stimulation index (ρ = −0.374, 95% CI −0.538 to −0.188, FDR q = 0.011) and IL-1β shutdown efficiency (ρ = −0.351, 95% CI −0.494 to −0.176, FDR q = 0.021). In resection-margin mucosa, CD4+ cell density correlated positively with IL-6 shutdown efficiency (ρ = 0.344, 95% CI 0.169 to 0.503, FDR q = 0.021) and IL-1β basal activity (ρ = 0.337, 95% CI 0.141 to 0.507, FDR q = 0.023). Representative scatter plots illustrating these systemic–local associations are shown in **Figure 5C**.

Together, these findings indicate that cytokine-response features measured in M-CSF-differentiated cultures derived from peripheral-blood CD14+ cells are associated with the local immune-cell context of CRC mucosa. The observed correlations link the MCP-1 response axis to local CD68+ cell density and connect IL-1β/IL-6 response indices predominantly with CD4+ cell densities in tumor-adjacent or resection-margin mucosa. These associations suggest partial correspondence between blood-derived ex vivo functional response features and mucosal immune-cell organization, without establishing the direction or mechanism of this relationship.

## 4 Discussion

In this study, we investigated CRC-associated immune remodeling using complementary blood-derived ex vivo and tissue-level readouts. M-CSF-differentiated cultures derived from peripheral-blood CD14+ cells showed selective alterations in IL-1β and IL-6 response indices, whereas tumor-adjacent and resection-margin mucosa demonstrated compartment-specific differences in CD20+ and CD68+ cell densities, coordinated cross-compartment immune-cell patterns, and grade-associated differences in selected immune-cell markers. Associations between cytokine-response indices and local immune-cell densities further suggested partial correspondence between these *ex vivo* functional features and mucosal immune-cell organization. A secondary exploratory analysis identified additional IL-1β and IL-6 response patterns according to distant metastasis status; however, the small number of patients with distant metastases precludes strong conclusions regarding metastasis-associated immune mechanisms.

A central feature of the present work is the use of dynamic cytokine-response indices rather than single static cytokine measurements. Static cytokine concentrations provide information about a particular experimental state, but they do not capture how cells respond, resolve, persist, or react to repeated stimulation in the sequential LPS stimulation model. By applying sequential LPS stimulation after M-CSF-driven differentiation of blood-derived CD14+ cells, we were able to distinguish basal activity, basal drift, primary LPS responsiveness, post-stimulation persistence, shutdown efficiency, re-induction capacity, and re-stimulation behavior. The combination of positive CD14+ cell selection, five-day M-CSF culture, medium replacement, and washing would be expected to preferentially retain cells of the monocyte–macrophage lineage, however, in the absence of post-differentiation phenotyping, the purity and precise differentiation state of the final cultures cannot be established. This approach allowed us to characterize immune responsiveness as a trajectory of functional states rather than as an isolated endpoint. In this context, the differences observed in CRC patients likely reflect altered regulatory properties of monocyte/macrophage-lineage cells, including changes in persistence and resolution of cytokine secretion after LPS stimulation.

This dynamic approach complements predominantly tissue-based studies of CRC immunity by providing a standardized ex vivo readout of cytokine-response properties retained after M-CSF differentiation of blood-derived CD14+ cells. Tumor-associated macrophages and other innate immune populations are important components of CRC progression and immune regulation, however, the present assay does not directly measure their function *in vivo*. Rather, our findings show that cytokine-response properties of M-CSF-differentiated CD14+ cell-derived cultures are altered in CRC and are associated with local mucosal immune-cell composition (8).

These findings can also be considered within the broader framework of myeloid-cell reprogramming and innate immune memory. CRC-associated transcriptional and metabolic programming of circulating monocytes has been previously reported (9, 15), while experimental studies indicate that tumor-associated systemic signals can epigenetically precondition peripheral monocytes before their entry into the tumor microenvironment. Repeated inflammatory stimulation can likewise establish persistent transcriptional and epigenetic states that modify responsiveness to subsequent LPS challenge (16). However, the present study did not assess epigenetic, transcriptional, or metabolic remodeling and therefore does not demonstrate trained immunity or inflammatory memory. Rather, the altered persistence, shutdown, and re-induction patterns observed here are functionally compatible with altered regulation of innate immune response states and provide a rationale for future mechanistic studies incorporating epigenomic and metabolic profiling.

The most prominent CRC-associated changes were observed for IL-1β and IL-6. For IL-1β, CRC patients showed higher post-washout persistence, reduced shutdown efficiency, reduced re-induction capacity, and a higher re-stimulation index. This pattern suggests that IL-1β regulation in CRC is not simply shifted toward globally higher or lower secretion, but is altered in its temporal control after LPS challenge. Increased persistence and reduced shutdown efficiency are consistent with impaired resolution of the first LPS-induced response, whereas the higher re-stimulation index suggests relative preservation of the second response compared with the primary response. Importantly, we deliberately refer to this parameter as a re-stimulation index rather than as a direct measure of “tolerance,” because ex vivo cytokine behavior in a simplified culture system should not be equated with systemic immune tolerance *in vivo*. Nevertheless, the observed pattern is compatible with altered post-stimulation control of IL-1β secretion in CRC-associated innate immune reprogramming.

The relevance of IL-1β to cancer inflammation is consistent with previous work showing that IL-1β signaling can contribute to tumor-promoting inflammatory networks, although its role is context-dependent and may differ across tumor types, cell sources, and disease stages. In CRC, IL-1-related signaling has also been implicated in tumor–stroma interactions, including inflammatory fibroblast programs linked to immune suppression and tumor growth. Our findings do not define the cellular source of IL-1β *in vivo*, but they indicate that M-CSF-differentiated cultures derived from peripheral-blood CD14+ cells from CRC patients display altered IL-1β response control after inflammatory stimulation (17).

The prominent alterations in IL-1β responsiveness also raise the possibility that inflammasome-dependent regulation contributes to the observed phenotype. Canonical inflammasome activation, particularly through NLRP3, leads to caspase-1-mediated processing of pro-IL-1β into its mature secreted form and can promote gasdermin D-dependent pyroptotic cell death (18). Altered NLRP3–caspase-1 signaling has been reported in colorectal cancer and may therefore represent one potential mechanism influencing IL-1β output in the present ex vivo model (19). However, this interpretation remains speculative. We did not assess NLRP3 or ASC expression, caspase-1 activation, gasdermin D cleavage, or pyroptotic cell death, and extracellular LPS stimulation alone does not specifically establish NLRP3 inflammasome activation. Thus, the observed IL-1β response patterns should not be interpreted as direct evidence of inflammasome activation or pyroptosis, but rather as a rationale for future mechanistic studies of this pathway.

IL-6 showed a different configuration of CRC-associated changes. CRC patients had lower basal IL-6 activity, but higher basal drift, stronger primary LPS response, and increased post-washout persistence. This indicates a dissociation between basal cytokine tone and inducible responsiveness. Reduced basal IL-6 activity in CRC cultures does not imply globally suppressed IL-6 biology; rather, it coexisted with enhanced response to LPS and greater persistence after stimulation. Such a pattern may reflect altered set-point regulation of CD14+ cell-derived cultures, in which the basal state and stimulated state are differentially affected. Together with the IL-1β findings, these data support the concept that CRC is associated with remodeling of dynamic cytokine responsiveness, particularly along inflammatory axes involved in innate immune activation and macrophage-related inflammatory signaling.

The prominence of IL-6 in our dynamic model is concordant with extensive evidence implicating IL-6/STAT3-related inflammatory signaling in CRC development and progression. Increased IL-6 expression has been associated with advanced disease and poorer survival in CRC, and IL-6/STAT3 signaling has been linked to tumor-cell survival, proliferation, invasion, angiogenesis, immune modulation, and therapy resistance. Our results complement this literature by showing that altered IL-6 regulation can also be detected as inducible and post-stimulation behavior in M-CSF-differentiated cultures derived from peripheral-blood CD14+ cells (12).

Notably, MCP-1 and IL-8 did not show comparable FDR-significant differences between CRC patients and comparison subjects in the primary between-group analysis. This negative result is informative because it argues against a nonspecific global shift in all measured cytokines. Instead, the disease-associated signal appeared concentrated in IL-1β and IL-6 dynamic indices. At the same time, MCP-1 became relevant in the systemic–local correlation analysis, where primary MCP-1 response was inversely associated with CD68+ cell density in tumor-adjacent mucosa. Thus, absence of a direct CRC-versus-comparison difference for MCP-1 does not exclude its biological relevance in the integrated systemic–local immune context.

The choice of the comparison group should be considered when interpreting the CRC-versus-comparison findings. Patients with non-malignant, non-incarcerated abdominal wall hernias were recruited within the same surgical setting and underwent the same preoperative blood-sampling and laboratory procedures as patients with CRC. The groups were also closely comparable in age and sex, and the principal between-group analyses were adjusted for these variables. This design reduced differences related to recruitment and preanalytical conditions, and blood collection before surgery excluded an acute postoperative inflammatory response as an explanation for the observed cytokine-response patterns.

Nevertheless, patients with abdominal wall hernias should not be regarded as immunologically equivalent to healthy volunteers. Unmeasured metabolic characteristics, comorbid conditions, or subclinical low-grade inflammation may have influenced basal and LPS-induced cytokine responses in the comparison group. Such factors could, for example, contribute to the relatively higher basal IL-6 activity observed in comparison subjects or modify post-washout IL-1β persistence, thereby either amplifying or attenuating the apparent CRC-associated differences. The present findings should therefore be interpreted as differences between patients with CRC and this specific non-malignant surgical comparison cohort, rather than as deviations from a population-based healthy reference.

The secondary exploratory analysis according to distant metastasis status identified differences in selected IL-1β and IL-6 response indices. Patients with distant metastases showed lower IL-1β basal activity together with higher basal drift, primary LPS response, persistence, re-induction capacity, and re-stimulation index. For IL-6, the presence of distant metastases was associated with higher basal activity and re-stimulation index but lower primary LPS response and shutdown efficiency. These patterns differed from those observed in the primary CRC-versus-comparison analysis, suggesting that the exploratory metastasis-associated findings were not simply an amplification of the general CRC-associated response profile. However, because only nine patients had distant metastases, the estimates are inherently imprecise and may be sensitive to sampling variability. These findings should therefore be regarded as preliminary and hypothesis-generating.

The observed associations can be considered in the broader context of myeloid and macrophage-related immune circuits implicated in CRC progression. Tumor-associated macrophages and recruited monocyte-lineage cells contribute to invasion, angiogenesis, immune suppression, and metastatic niche formation. Nevertheless, the present assay did not assess circulating monocytes directly, tumor-associated macrophages, metastatic lesions, or pre-metastatic niches. Instead, it measured cytokine-response potential after standardized M-CSF-driven differentiation and sequential LPS stimulation of blood-derived CD14+ cells. The results are therefore compatible with an association between distant metastasis status and the functional response phenotype retained by these *ex vivo* cultures, but they do not demonstrate altered myeloid function *in vivo* or define a mechanism of metastatic dissemination (8).

The metastasis-related contribution of this study is consequently exploratory. Sequential LPS stimulation provided response-state variables including persistence, shutdown efficiency, re-induction capacity, and re-stimulation behavior that are not captured by static cytokine measurements. These variables may inform future studies of immune response phenotypes associated with gastrointestinal cancer dissemination, but their reproducibility, discriminatory performance, and prognostic relevance remain to be established. Validation will require larger independent cohorts with adequate representation of metastatic disease, longitudinal sampling, and integration with primary and metastatic tissue profiling and more detailed immune-cell characterization.

The local mucosal compartment showed its own distinct pattern of immune remodeling. CD20+ cells were enriched in tumor-adjacent mucosa, whereas CD68+ cells were markedly enriched in resection-margin mucosa. These findings indicate that the local immune landscape differs between tissue compartments and that tumor-adjacent mucosa cannot be treated as immunologically equivalent to resection-margin mucosa. The CD20+ enrichment near the tumor may reflect local organization of B-cell-associated immune responses in the tumor-adjacent mucosal environment. In contrast, the higher CD68+ cell density in resection-margin mucosa suggests that macrophage-lineage cell accumulation is not restricted to the immediate tumor-adjacent compartment and may reflect broader mucosal immune activation or tissue-level inflammatory remodeling. Although previous studies have emphasized macrophage enrichment and prognostic relevance at the tumor invasive front in colorectal cancer, particularly using CD68-based macrophage assessment (20), our analysis addressed a different spatial contrast: tumor-adjacent mucosa versus resection-margin mucosa. These interpretations should remain cautious, because CD68 is a broad macrophage-lineage marker and does not distinguish functional macrophage states.

The CD20+ finding is consistent with increasing recognition that B cells are relevant components of CRC immunity. Tumor-infiltrating CD20+ B lymphocytes have been associated with favorable prognosis in CRC and may interact functionally or spatially with CD8+ T-cell infiltration, although the role of B-cell subsets in CRC is heterogeneous and context-dependent. Our study did not phenotype B-cell subsets or assess tertiary lymphoid structures or germinal-center-associated markers. Therefore, the observed enrichment of CD20+ cells cannot be interpreted as evidence of tertiary lymphoid structure formation. It nevertheless supports the presence of B-cell-related immune organization within the tumor-adjacent mucosal immune landscape (21).

Despite the compartment-specific redistribution of selected immune-cell populations, matched marker densities were positively correlated between tumor-adjacent and resection-margin mucosa for all analyzed immune-cell subsets. The strongest cross-compartment correlation was observed for CD8+ cells, suggesting that interindividual differences in cytotoxic T-cell density are preserved across both mucosal compartments. Similar, although weaker, correlations were observed for CD20+, CD56+, CD4+, and CD68+ cells. This indicates that local immune-cell composition is not entirely compartment-autonomous. Rather, each patient appears to have a partly coordinated mucosal immune background on which compartment-specific changes are superimposed. This distinction is important: paired differences describe compartmental redistribution, whereas cross-compartment correlations describe whether interindividual immune-cell ranking is preserved across mucosal sites.

Previous studies have shown that morphologically normal or tumor-distant colorectal mucosa in CRC patients may have an altered immune profile and should not be regarded as an immunologically inert comparator. Strasser et al. demonstrated immunological differences between CRC tissue and tumor-distant normal mucosa (22), while studies of Lynch syndrome and MSI CRC further showed that immune-cell densities and immune-related gene expression in tumor-distant normal mucosa differ according to cancer status and molecular background (13). More recent transcriptomic and spatial analyses also support the presence of field-like biological variability in non-tumor colorectal mucosa of CRC patients (14). Our data extend these observations by directly comparing tumor-adjacent mucosa with resection-margin mucosa and by distinguishing two complementary phenomena: compartment-specific redistribution of selected immune-cell populations and matched-marker cross-compartment coupling. Thus, resection-margin mucosa in CRC should be interpreted not simply as “normal control tissue,” but as part of a broader mucosal immune field that may retain patient-specific immune-cell patterns.

Histological grade was positively associated with CD68+ cell density in both tumor-adjacent and resection-margin mucosa and with CD4+ cell density in resection-margin mucosa. These associations suggest that poorer tumor differentiation is accompanied by broader local immune remodeling involving macrophage-lineage and CD4+ cell compartments. The presence of grade-associated correlations in resection-margin mucosa is particularly notable, because it suggests that immune alterations linked to tumor aggressiveness may extend beyond the immediate tumor-adjacent compartment. However, these findings should not be interpreted as evidence that CD68+ or CD4+ cells directly drive dedifferentiation. Rather, they support an association between histological aggressiveness and the local mucosal immune context.

The association between CD68+ cell density and higher histological grade should be interpreted in light of the functional and metabolic heterogeneity of macrophages in the tumor microenvironment (23). TAMs can support tumor progression through cytokine and chemokine secretion, matrix remodeling, angiogenesis, immune suppression, and metastatic niche formation, but their prognostic impact may depend on anatomical compartment, marker combination, macrophage polarization state, and tumor context (24). The use of CD68 alone therefore provides information on macrophage-lineage density but not on macrophage functional state, reinforcing the need for more granular phenotyping in future studies (25).

The cross-domain correlation analysis identified an inverse association between MCP-1 primary LPS response in the ex vivo culture model and CD68+ cell density in tumor-adjacent mucosa. Given the role of MCP-1/CCL2 in monocyte recruitment and macrophage-related inflammatory networks, this association is biologically relevant; however, its interpretation is limited by the nature of the CD68 marker. CD68 identifies cells of the macrophage lineage but does not distinguish their functional states or activation programs. Accordingly, the observed correlation should be interpreted as an association between ex vivo MCP-1 responsiveness and local CD68+ cell density, rather than as evidence that a specific macrophage phenotype is linked to altered MCP-1 responsiveness. The inverse direction may reflect shared or unmeasured factors influencing both features, but the present cross-sectional data cannot establish the direction or mechanism of this relationship.

CD4+ cell densities were also associated with several IL-1β and IL-6 response indices. In tumor-adjacent mucosa, CD4+ cell density correlated inversely with the IL-6 re-stimulation index and IL-1β shutdown efficiency, whereas in resection-margin mucosa CD4+ cell density correlated positively with IL-6 shutdown efficiency and IL-1β basal activity. These findings indicate that cytokine-response features measured in M-CSF-differentiated cultures derived from peripheral-blood CD14+ cells are related to the local CD4+ cell context, with the direction of the associations depending on tissue compartment and cytokine-response index. As with the MCP-1–CD68 association, these correlations demonstrate correspondence between ex vivo functional and tissue-level immune features but do not establish a causal or directional relationship. Because CD4 was assessed as a single immunohistochemical marker, these associations cannot be assigned to specific CD4+ T-cell subsets, including regulatory T cells. Additional markers such as FOXP3 and CD25 would be required for more detailed functional phenotyping.

Recent single-cell RNA sequencing and spatial profiling studies have substantially refined the view of the colorectal cancer immune microenvironment by revealing marked heterogeneity among myeloid and lymphoid populations and by identifying spatially organized multicellular immune niches (26, 27). In this context, our immunohistochemical assessment provides a lower-dimensional but spatially anchored measure of selected immune-cell populations across matched mucosal compartments, whereas the ex vivo stimulation model adds functional information on cytokine-response regulation that is not directly captured by static tissue profiling. Integration of these complementary approaches in future studies could help identify the specific myeloid and lymphoid cell states, stromal interactions, and spatial niches associated with the cytokine-response phenotypes observed here.

From a translational perspective, dynamic cytokine-response profiling may provide information complementary to conventional clinicopathological and molecular characteristics by capturing how blood-derived myeloid-lineage cultures respond across sequential inflammatory states. If reproducible in independent prospective cohorts, such functional response patterns could potentially be explored for patient stratification, longitudinal treatment monitoring, or assessment of recurrence and metastatic risk. However, the present study was not designed to establish diagnostic or predictive performance. We did not evaluate longitudinal changes, recurrence or survival outcomes, treatment response, or response to immunotherapy, and the exploratory metastasis analysis included only nine patients with distant metastases. Accordingly, the identified cytokine-response indices should currently be regarded as research-level functional phenotypes rather than clinically validated biomarkers. Clinical implementation would require assay standardization, assessment of analytical and biological reproducibility, definition of clinically relevant thresholds, and prospective validation against predefined clinical endpoints in independent cohorts.

Taken together, the data indicate that CRC is associated with complementary alterations in blood-derived ex vivo cytokine responsiveness and local mucosal immune-cell organization. M-CSF-differentiated cultures derived from peripheral-blood CD14+ cells showed altered IL-1β and IL-6 response patterns, whereas tumor-adjacent and resection-margin mucosa demonstrated compartment-specific immune-cell redistribution, cross-compartment coordination of immune-cell densities, and grade-associated differences in selected immune-cell markers. The systemic - local correlations further suggest that cytokine-response features measured in these *ex vivo* cultures are related to mucosal immune-cell composition, although the direction and biological basis of these relationships cannot be determined from the present observational design. The exploratory analysis according to distant metastasis status identified additional IL-1β and IL-6 response patterns that warrant validation in larger cohorts. These findings provide a basis for subsequent studies integrating functional blood-derived assays with detailed phenotyping of primary tumors, metastatic lesions, and the broader immune microenvironment.

Several limitations should be considered.

First, this was an observational study, and the results do not establish causal relationships between blood-derived ex vivo cytokine-response features, local immune-cell composition, and tumor progression.

Second, the comparison group comprised patients with non-malignant, non-incarcerated abdominal wall hernias rather than population-based healthy volunteers. Although blood was collected before surgery under the same preanalytical conditions in both groups, and the groups were closely comparable in age and sex, unmeasured metabolic characteristics, comorbidities, and subclinical low-grade inflammation may have affected basal or LPS-induced cytokine responses. Consequently, the observed differences should be interpreted as CRC-associated contrasts relative to this specific non-malignant surgical cohort, rather than as deviations from a healthy reference population.

Third, the exploratory analysis according to distant metastasis status was based on only nine patients with distant metastases. Consequently, effect estimates may be unstable, statistical power was limited, and the observed associations should not be interpreted as validated biomarkers, predictors of metastatic progression, or evidence of a distinct metastatic immune phenotype. These findings require confirmation in larger independent cohorts with adequate representation of metastatic disease. Fourth, metastatic tissue and pre-metastatic organs were not examined, therefore, the study cannot define immune mechanisms operating within metastatic lesions or establish a direct relationship between the ex vivo cytokine-response patterns and immune events at distant sites. Given the small number of patients with distant metastases, ROC analysis, decision-curve analysis, and internal validation of metastatic-disease discrimination were not performed, because the resulting performance estimates would be imprecise and potentially misleading.

Fifth, cytokine responses were measured after a five-day M-CSF-driven differentiation period and therefore cannot be interpreted as direct functional readouts of freshly isolated circulating monocytes. The observed response patterns may reflect both donor-cell properties retained during culture and changes acquired under the experimental conditions. Post-differentiation cellular phenotype and purity were not directly characterized, and the simplified culture model cannot reproduce the full complexity of *in vivo* immune regulation.

Sixth, the cytokine panel was limited to IL-1β, IL-6, IL-8, and MCP-1, and the immunohistochemical panel was limited to CD4, CD8, CD20, CD56, and CD68. Other relevant mediators, including TNF-α, IL-10, IFN-γ, IL-17A, CXCL9/CXCL10, GM-CSF, and TGF-β, were not assessed. Their inclusion could have provided a broader view of inflammatory, regulatory, and lymphoid-associated immune programs. The present measurements therefore provide a focused but incomplete view of blood-derived ex vivo and local immune features. Additional circulating or soluble immune biomarkers, including soluble IL-6 receptor, soluble CD163, osteopontin, S100A8/A9, HMGB1, and circulating myeloid-derived suppressor cells, were not evaluated. Their inclusion in future studies could help place the observed ex vivo cytokine-response patterns within a broader systemic inflammatory and myeloid-regulatory context. Molecular characterization was also limited to KRAS, NRAS, and BRAF mutation status. Microsatellite instability/mismatch-repair status, Consensus Molecular Subtypes, tumor mutational burden, and immune-checkpoint expression were not assessed. Integration of these molecular and immune features could provide a more comprehensive framework for interpreting interindividual variation in ex vivo cytokine responsiveness and local immune-cell organization.

Seventh, longitudinal follow-up data were not available; therefore, recurrence-free, disease-free, and overall survival could not be evaluated. Consequently, the present study cannot determine whether the identified ex vivo cytokine-response patterns or mucosal immune-cell features have prognostic significance. Future studies with longitudinal follow-up will be required to assess their associations with recurrence and survival outcomes.

Eighth, this was a single-center study of participants recruited in a surgical setting. Therefore, the generalizability of the findings to broader CRC populations, particularly patients managed in non-surgical settings or at other institutions, may be limited.

Finally, although multiple testing was addressed using FDR correction and adjusted models were applied where appropriate, some analyses, particularly subgroup and correlation analyses, should be considered exploratory.

## 5 Conclusion

This study demonstrates that CRC is associated with altered cytokine-response patterns in M-CSF-differentiated cultures derived from peripheral-blood CD14+ cells, with the most consistent differences involving IL-1β and IL-6. Tumor-adjacent and resection-margin mucosa also showed compartment-specific differences in CD20+ and CD68+ cell densities, coordinated immune-cell patterns across mucosal compartments, and grade-associated differences in selected immune-cell markers. Associations between ex vivo cytokine-response indices and local immune-cell densities suggest partial coupling between blood-derived functional response features and mucosal immune-cell organization, although their direction and biological basis remain uncertain. The exploratory comparison according to distant metastasis status identified additional IL-1β and IL-6 response patterns, but these findings are based on only nine patients with distant metastases and require validation in larger, adequately powered cohorts. Future studies should integrate longitudinal functional assays with detailed phenotyping of primary tumors, metastatic lesions, and circulating immune-cell populations.

## Data Availability

The raw data supporting the conclusions of this article will be made available by the authors, without undue reservation.

## Glossary

A260/A280: absorbance ratio at 260/280 nm (spectrophotometric DNA purity metric)
AI: artificial intelligence
AJCC: American Joint Committee on Cancer
B24: basal cytokine secretion after 24 h
B48: basal cytokine secretion after 48 h
BRAF: B-Raf proto-oncogene, serine/threonine kinase
CCL2: C-C motif chemokine ligand 2
CD4+: cluster of differentiation 4-positive cells
CD8+: cluster of differentiation 8-positive cells
CD14+: cluster of differentiation 14-positive cells
CD20+: cluster of differentiation 20-positive cells
CD56+: cluster of differentiation 56-positive cells
CD68+: cluster of differentiation 68-positive cells
CI: confidence interval
CRC: colorectal cancer
CXCL8: C-X-C motif chemokine ligand 8
DNA: deoxyribonucleic acid
EDTA: ethylenediaminetetraacetic acid
ELISA: enzyme-linked immunosorbent assay
FDR: false discovery rate
FFPE: formalin-fixed, paraffin-embedded
G1: grade 1
G2: grade 2
G3: grade 3
HIV: human immunodeficiency virus
IL: interleukin
IL-1β: interleukin-1 beta
IL-1F2: interleukin-1 family member 2
IL-6: interleukin-6
IL-8: interleukin-8
IQR: interquartile range
KRAS: Kirsten rat sarcoma viral oncogene homolog
log1p: natural logarithm of one plus x transformation
LPS: lipopolysaccharide
LPS1: cytokine response after the first LPS stimulation
LPS2: cytokine response after the second LPS stimulation
M-CSF: macrophage colony-stimulating factor
M0: absence of distant metastases
M1: presence of distant metastases
MCP-1: monocyte chemoattractant protein-1
MSI: microsatellite instability
NRAS: neuroblastoma RAS viral oncogene homolog
PCR: polymerase chain reaction
PERSIST: post-stimulation persistence after LPS washout without repeated stimulation
RPMI-1640: Roswell Park Memorial Institute 1640 medium
SD: standard deviation
STAT3: signal transducer and activator of transcription 3
TAMs: tumor-associated macrophages
TNF-α: tumor necrosis factor alpha
TNM: Tumor–Node–Metastasis staging system
UICC: Union for International Cancer Control
β: beta coefficient
p: probability value
q: FDR-adjusted p value
ρ: Spearman correlation coefficient
