# Supplementary material for: Systemic–local immune remodeling in colorectal cancer: CD14+ cell-derived cytokine responsiveness, mucosal immune-cell organization, and exploratory metastasis-associated patterns
Source: Front Immunol. 2026 Sep 9;17:1918710. doi: 10.3389/fimmu.2026.1918710 (PMC13597458; doi:10.3389/fimmu.2026.1918710)
Supplement: Supplementary file 1 [file DataSheet1.pdf]

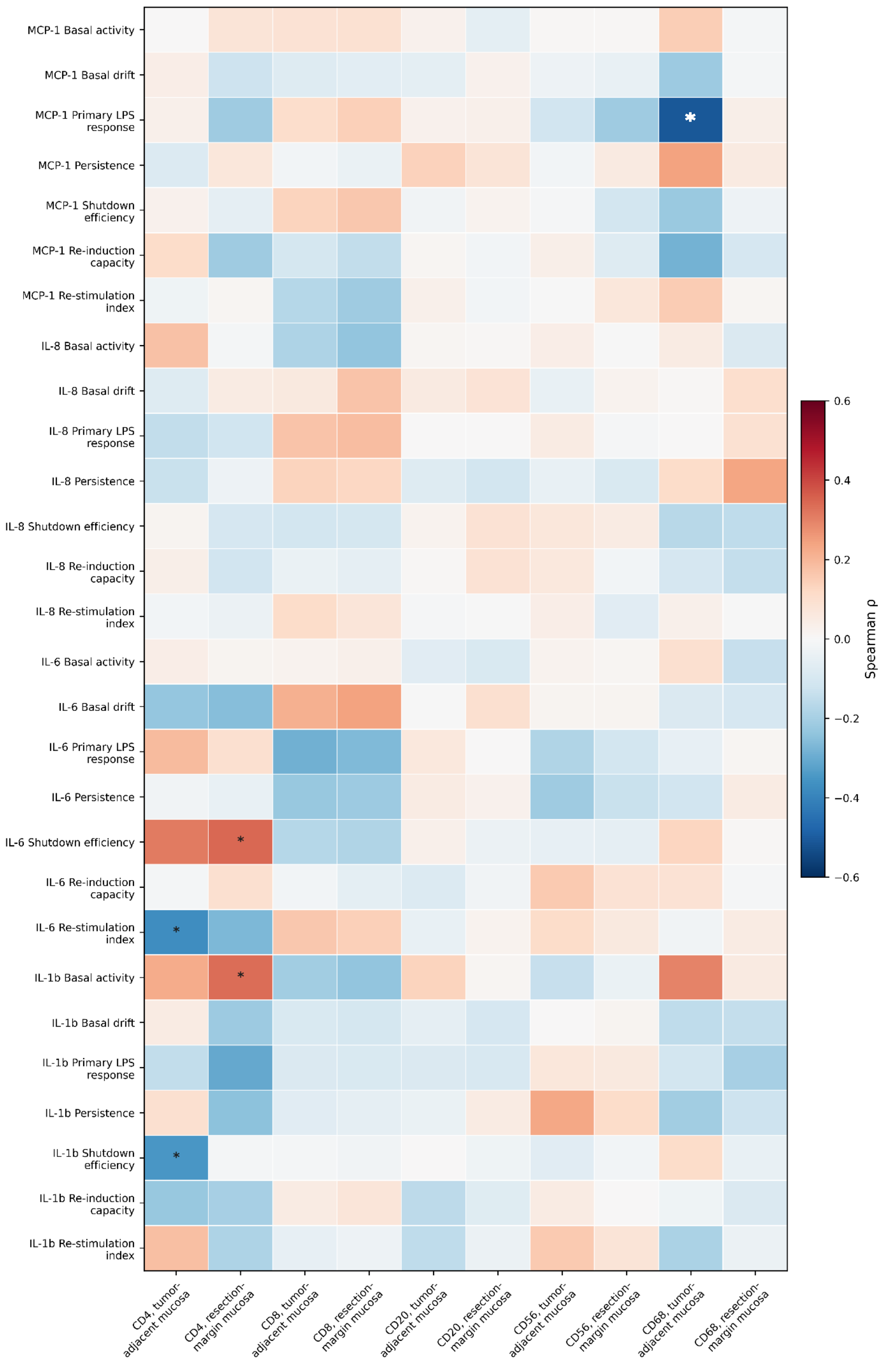

Supplementary Figure S1. Full systemic–local correlation heatmap.
Heatmap showing Spearman correlations between all dynamic cytokine-response indices and local immune-cell densities in tumor-adjacent and resection-margin mucosa. Dynamic cytokine-response indices were calculated from log1p-transformed cytokine concentrations. Local immune-cell densities are expressed as cells per visual field at ×400 magnification. Cell color indicates Spearman correlation coefficient (ρ), with the color scale truncated at −0.6 to +0.6 for visualization. Asterisks indicate correlations significant after Benjamini–Hochberg FDR correction across the full systemic–local correlation matrix (q < 0.05). Full numerical results are provided in Supplementary Table S5.

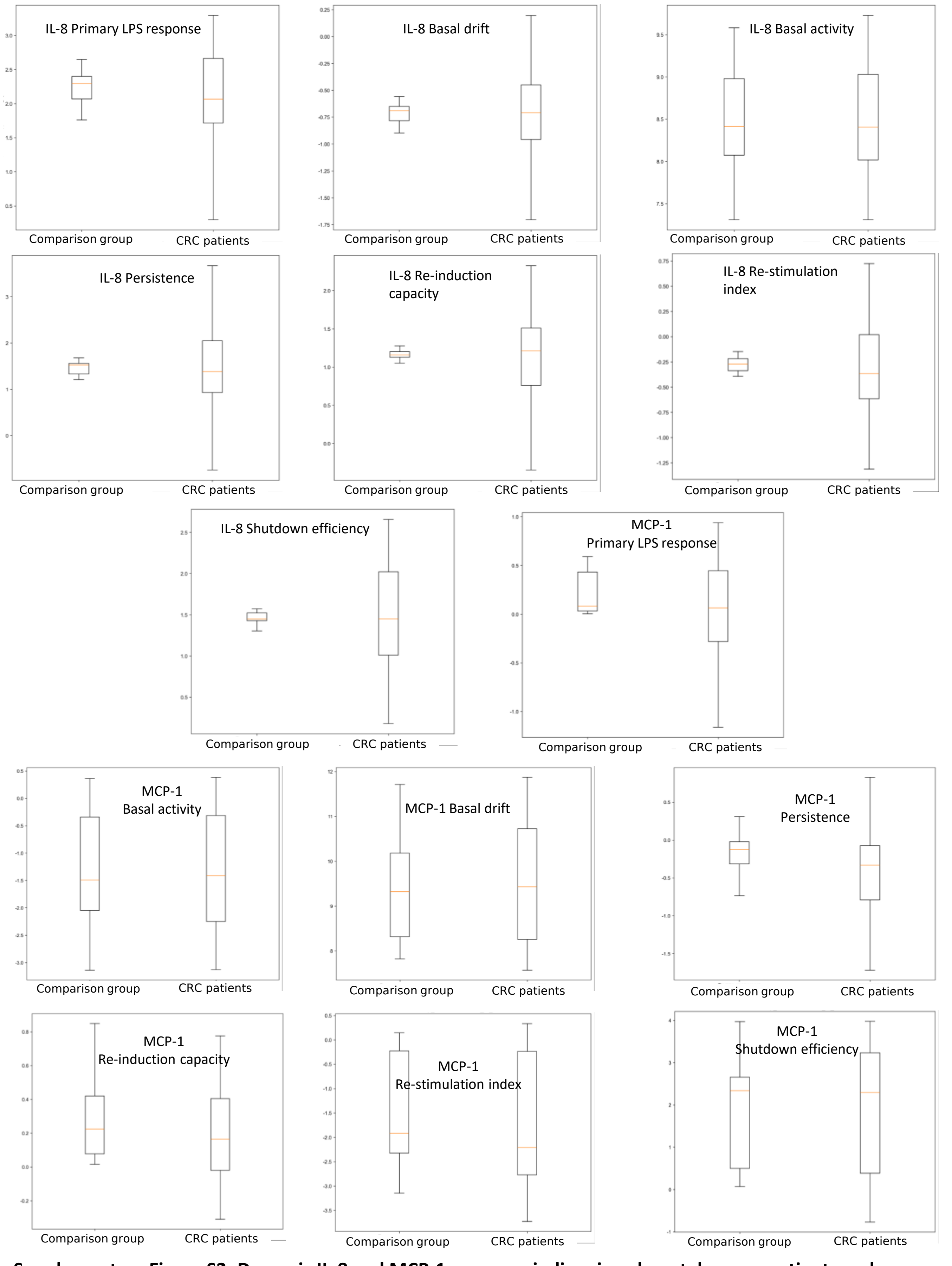

Supplementary Figure S2. Dynamic IL-8 and MCP-1 response indices in colorectal cancer patients and comparison subjects.

Boxplots show the distributions of IL-8 and MCP-1 dynamic cytokine-response indices in the comparison group and in patients with colorectal cancer (CRC). All indices were calculated from log1p-transformed cytokine concentrations. Boxplots indicate the median and interquartile range, with whiskers showing the data range according to the plotting convention. No IL-8 or MCP-1 dynamic index showed significant differences between CRC patients and comparison subjects in the corresponding between-group analysis.
