# Supplementary material for: Systemic–local immune remodeling in colorectal cancer: CD14+ cell-derived cytokine responsiveness, mucosal immune-cell organization, and exploratory metastasis-associated patterns
Source: Front Immunol. 2026 Sep 9;17:1918710. doi: 10.3389/fimmu.2026.1918710 (PMC13597458; doi:10.3389/fimmu.2026.1918710)
Supplement: Supplementary file 2 [file Table3.docx]

STROBE Statement—Checklist of items that should be included in reports of ***cross-sectional studies***

*Reporting locations refer to the final revised manuscript.*

|  | **Item No** | **Recommendation** | **Reported on page / section** |
| --- | --- | --- | --- |
| **Title and abstract** | 1 | (*a*) Indicate the study’s design with a commonly used term in the title or the abstract | p. 1, Abstract |
|  |  | (*b*) Provide in the abstract an informative and balanced summary of what was done and what was found | p. 1, Abstract |
| **Introduction** | | | |
| Background/rationale | 2 | Explain the scientific background and rationale for the investigation being reported | pp. 2–3, Introduction |
| Objectives | 3 | State specific objectives, including any prespecified hypotheses | pp. 3–4, final paragraph of Introduction |
| **Methods** | | | |
| Study design | 4 | Present key elements of study design early in the paper | p. 4, Section 2.1 (Study design and participants) |
| Setting | 5 | Describe the setting, locations, and relevant dates, including periods of recruitment, exposure, follow-up, and data collection | p. 4, Section 2.1 (single-center setting; Moscow; May–December 2025) |
| Participants | 6 | (*a*) Give the eligibility criteria, and the sources and methods of selection of participants | p. 4, Section 2.1 (eligibility criteria; consecutive recruitment) |
| Variables | 7 | Clearly define all outcomes, exposures, predictors, potential confounders, and effect modifiers. Give diagnostic criteria, if applicable | pp. 4–9, Sections 2.2–2.9; Table 1, p. 24 |
| Data sources/ measurement | 8* | For each variable of interest, give sources of data and details of methods of assessment (measurement). Describe comparability of assessment methods if there is more than one group | p. 4, Section 2.1; pp. 5–8, Sections 2.3–2.8 |
| Bias | 9 | Describe any efforts to address potential sources of bias | p. 4, Section 2.1; p. 5, Section 2.3; pp. 8–9, Section 2.9; p. 15 and pp. 18–20, Discussion/Limitations |
| Study size | 10 | Explain how the study size was arrived at | p. 9, Section 3.1 (study size determined by eligible participants enrolled during the predefined recruitment period; no a priori power calculation) |
| Quantitative variables | 11 | Explain how quantitative variables were handled in the analyses. If applicable, describe which groupings were chosen and why | pp. 7–9, Sections 2.7 and 2.9; Table 1 |
| Statistical methods | 12 | (*a*) Describe all statistical methods, including those used to control for confounding | pp. 8–9, Section 2.9 |
|  |  | (*b*) Describe any methods used to examine subgroups and interactions | pp. 8–9, Section 2.9 (M0/M1 and mutation-specific subgroup analyses) |
|  |  | (*c*) Explain how missing data were addressed | p. 8, Section 2.9 (no imputation; available/complete observations; pairwise-complete correlations) |
|  |  | (*d*) If applicable, describe analytical methods taking account of sampling strategy | Not applicable — no complex sampling strategy was used. |
|  |  | (*e*) Describe any sensitivity analyses | Not applicable — no formal sensitivity analyses were performed. |
| **Results** | | | |
| Participants | 13* | (a) Report numbers of individuals at each stage of study—eg numbers potentially eligible, examined for eligibility, confirmed eligible, included in the study, completing follow-up, and analysed | p. 9, Section 3.1; Table 2, pp. 24–26. A total of 126 participants were included and analyzed. |
|  |  | (b) Give reasons for non-participation at each stage | Not available — a formal screening log was not maintained, so numbers/reasons for non-enrolment cannot be reported. |
|  |  | (c) Consider use of a flow diagram | Not used. A flow diagram was not constructed because screening-stage counts were not available. |
| Descriptive data | 14* | (a) Give characteristics of study participants (eg demographic, clinical, social) and information on exposures and potential confounders | p. 9, Section 3.1; Table 2, pp. 24–26 |
|  |  | (b) Indicate number of participants with missing data for each variable of interest | p. 8, Section 2.9 (no imputation; analyses based on available/complete observations); Table 2, pp. 25–26 (available denominators reported for clinicopathological variables; tumor sidedness was classifiable in 101/106 CRC patients). |
| Outcome data | 15* | Report numbers of outcome events or summary measures | pp. 10–12, Sections 3.2–3.5; Table 3; Figures 2–5; Supplementary Tables S1–S6 |
| Main results | 16 | (*a*) Give unadjusted estimates and, if applicable, confounder-adjusted estimates and their precision (eg, 95% confidence interval). Make clear which confounders were adjusted for and why they were included | pp. 8–10, Section 2.9 and Section 3.2; Table 3, pp. 27–28. Unadjusted comparisons, FDR-adjusted p values, and age-/sex-adjusted β estimates with 95% CIs are reported. |
|  |  | (*b*) Report category boundaries when continuous variables were categorized | Not applicable — continuous variables were not categorized in the reported analyses. |
|  |  | (*c*) If relevant, consider translating estimates of relative risk into absolute risk for a meaningful time period | Not applicable — no relative-risk estimates over a defined follow-up period were calculated. |
| Other analyses | 17 | Report other analyses done—eg analyses of subgroups and interactions, and sensitivity analyses | pp. 10–12, Sections 3.3–3.5; Supplementary Tables S2, S4–S6 |
| **Discussion** | | | |
| Key results | 18 | Summarise key results with reference to study objectives | p. 13, opening paragraphs of Discussion; p. 20, Conclusion |
| Limitations | 19 | Discuss limitations of the study, taking into account sources of potential bias or imprecision. Discuss both direction and magnitude of any potential bias | pp. 18–20, Limitations |
| Interpretation | 20 | Give a cautious overall interpretation of results considering objectives, limitations, multiplicity of analyses, results from similar studies, and other relevant evidence | pp. 13–20, Discussion and Conclusion |
| Generalisability | 21 | Discuss the generalisability (external validity) of the study results | p. 19, Limitations (single-center surgical cohort; external validity/generalizability) |
| **Other information** | | | |
| Funding | 22 | Give the source of funding and the role of the funders for the present study and, if applicable, for the original study on which the present article is based | p. 21, Funding (funding sources and statement that funders had no role in design, data collection/analysis, interpretation, or publication decision) |

*Give information separately for exposed and unexposed groups.

**Note:** An Explanation and Elaboration article discusses each checklist item and gives methodological background and published examples of transparent reporting. The STROBE checklist is best used in conjunction with this article (freely available on the Web sites of PLoS Medicine at http://www.plosmedicine.org/, Annals of Internal Medicine at http://www.annals.org/, and Epidemiology at http://www.epidem.com/). Information on the STROBE Initiative is available at www.strobe-statement.org.
